# Supplementary material for: Mechanisms of deformation and failure in colluvial slope under artificial surcharge loading
Source: PLoS One. 2026 Jan 5;21(1):e0339772. doi: 10.1371/journal.pone.0339772 (PMC12768376; doi:10.1371/journal.pone.0339772)
Supplement: S1 Appendix — (DOCX) [file pone.0339772.s001.docx]

**Appendix A**

**Soil Physical Properties Testing Procedure**

To support field investigations in the study area and facilitate subsequent model testing and numerical simulation, laboratory geotechnical tests are required to determine the fundamental physical and mechanical properties of the soil. Precise measurement of the landslide soil’s physical and mechanical parameters is essential for understanding its formation mechanisms, deformation behavior, and stability. These parameters directly influence the accuracy of numerical simulation analyses and the effectiveness of engineering prevention and mitigation measures. Therefore, tests on the basic physical and mechanical properties were conducted using soil samples collected from the study area.

1. **Ring Cutter Test**

The natural density was determined by collecting soil samples with a ring cutter and measuring their mass and volume.

**Testing Equipment**

The ring cutter (volume 200 cm³), ring‑cutter cover plate, ring‑cutter base plate, small spatula, precision balance (accuracy 0.01 g), and drying oven are presented in [Fig 1](#f1).


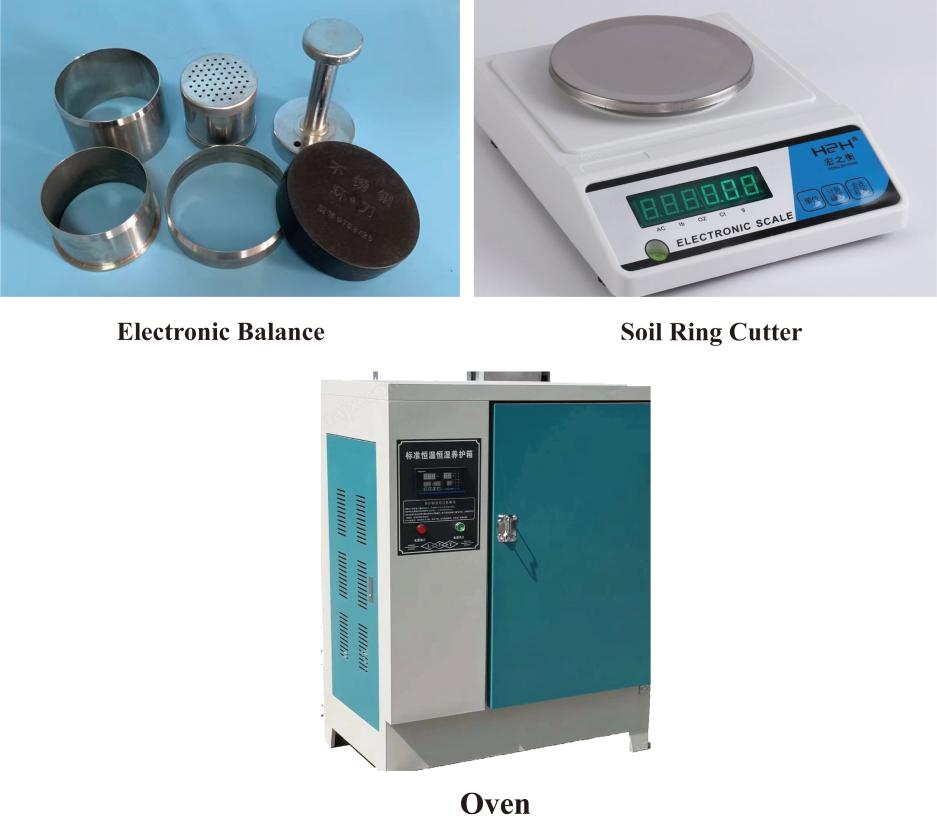


**Fig 1. Ring cutter, laboratory balance, and drying oven**

**Experimental Procedures**

Weigh the empty ring cutter and record its mass as M1

Fill the ring cutter with soil until the soil surface slightly exceeds the upper rim of the cutter.

Use a spatula to trim the soil at both ends of the ring cutter so that the soil surface is flush with the cutter edges.

Cover both ends of the ring cutter, weigh it again, and record the mass as M2

Calculate the natural density as follows:

$$\rho=\frac{M_{2}-M_{1}}{200}$$

A portion of the excavated soil was collected and placed in a moisture‑content container. The container was then placed in an oven and dried at 105–110 °C for 1 h. The mass of the container with wet soil was recorded as mw, and the mass after drying was recorded as md. The water content was calculated using the following equation:

$$\omega=\frac{m_{w}-m_{d}}{m_{d}}$$

1. **Proctor compaction testing**

The laboratory Proctor compaction test is a standard geotechnical method used to determine the maximum dry density and optimum moisture content of a soil specimen. By varying the water content of the soil samples, the moisture content and dry density of each specimen are measured, and a relationship curve is plotted with moisture content on the horizontal axis and dry density on the vertical axis. The peak of this curve represents the optimum moisture content on the horizontal axis and the maximum dry density on the vertical axis. The compaction energy applied in this test is approximately 2684.9 kJ/m³. A total of 15 specimens were prepared using the dry preparation method.

**Testing Equipment**

In this compaction test, a JS30‑5 heavy‑duty compaction apparatus designed for coarse‑grained soils was employed (as shown in [Fig. 2](#f2)). The hammer has a mass of 15.5 kg and a drop height of 600 mm. The compaction mold has an inner diameter of 300 mm and a height of 288 mm.

| 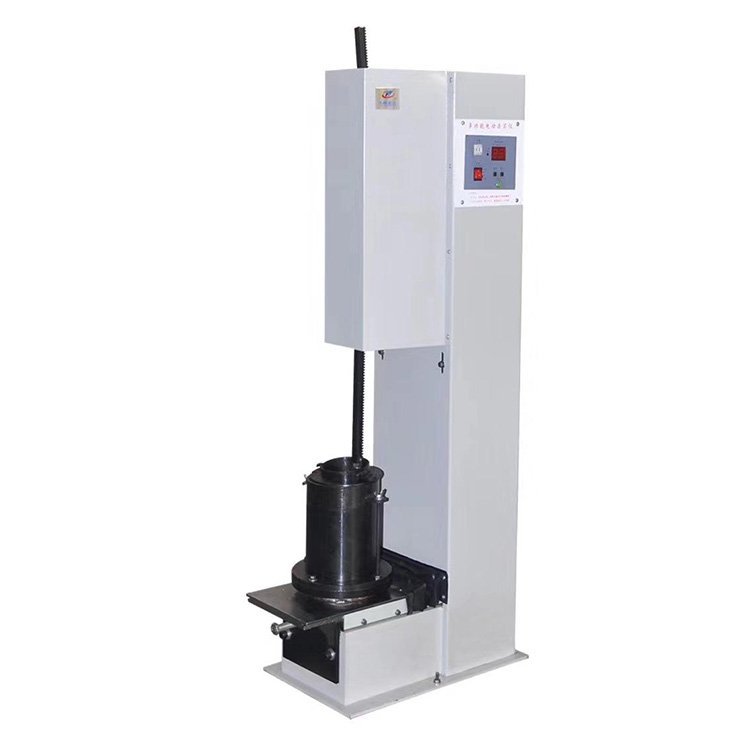 |
| --- |
| **Fig 2. Figure of the JS30‑5 heavy‑duty compaction apparatus used for coarse‑grained soils** |

**Specimen preparation**

The test materials were prepared using the dry method. A portion of the air‑dried soil was sieved to remove particles larger than 60 mm. Based on the particle‑size group proportions obtained from the sieve retention test, the mass of each particle‑size group was measured for each specimen. Five specimens were prepared for each of the three soil types, resulting in a total of 15 specimens. The moisture content of one selected specimen was measured. The optimum moisture content was estimated from the soil’s plastic limit. Subsequently, 15 specimens with different moisture contents were prepared, with the moisture‑content difference between adjacent specimens maintained at approximately 2%.

**Experimental Procedures**

（1） Instrument preparation: First, ensure that the compaction apparatus is securely fixed to a rigid base, with the compaction mold accurately connected to the base plate, and then install the guide sleeve. A uniform layer of petroleum jelly is subsequently applied to the inner wall and bottom surface of the compaction mold to reduce end‑effect influences.

（2） Soil placement and compaction: Weigh the soil sample and place it into the compaction mold in layers. Care must be taken to prevent the segregation of coarse particles and to maintain similar layer thicknesses. After each layer is compacted, the surface should be scarified. At the end of compaction, the specimen must not extend more than 15 mm above the top of the compaction mold.

（3） Post‑compaction handling: After compaction, remove the guide sleeve. Excess soil at the top of the mold is first trimmed to produce a level surface. The base plate is then detached, and the outer surface of the compaction mold is carefully cleaned. Finally, the combined mass of the compaction mold and soil specimen is measured with an accuracy of 50 g.

（4） Moisture‑content determination: A soil extruder is used to push the specimen out of the mold, and 5 kg of soil is collected from the middle portion of the specimen for moisture‑content testing. The difference in moisture content between the two test samples must not exceed 1%.

**3. Large‑scale triaxial shear testing for coarse‑grained soils**

Large‑scale triaxial testing of coarse‑grained soils is a laboratory method in which soil specimens are subjected to controlled loading using specialized triaxial testing equipment. By applying confining pressure and axial stress, the test evaluates the soil’s shear strength, deformation characteristics, and pore‑water pressure response under various stress conditions. The results provide essential soil parameters for subsequent research.

This study examines the mechanical response of the soil under unconsolidated–undrained (UU) conditions using a large‑scale triaxial testing system. Given that cobble‑containing soils, as a type of coarse‑grained soil, typically experience in‑situ engineering stresses ranging from 100 to 400 kPa, and considering both equipment capacity and prior testing experience, confining pressures of 100, 200, 300, and 400 kPa were selected. The specimens were prepared with a height‑to‑diameter ratio of 2:1 and tested under axial‑displacement control at a loading rate of 3.0 mm/min, with the drainage valve kept closed throughout the entire test. A total of 12 specimens were collected from the study area and prepared for testing.

This study examines the mechanical response of the soil under unconsolidated–undrained (UU) conditions using a large‑scale triaxial testing system. Considering that cobble‑containing soils, as a type of coarse‑grained soil, typically experience in‑situ engineering stresses between 100 and 400 kPa, and taking into account equipment capacity and experience from similar tests, confining pressures of 100, 200, 300, and 400 kPa were selected. The specimens were prepared as standard samples with dimensions of φ300 × 600 mm, corresponding to a height‑to‑diameter ratio of 2:1, and were tested under axial‑displacement control at a loading rate of 3.0 mm/min, with the drainage valve kept closed throughout the entire test. A total of four specimens were prepared.

**Testing Equipment**

The experimental apparatus used in this study was a VJT5025‑GDS large‑scale triaxial testing machine imported from the United Kingdom (as shown in [Fig  3](#f3)).


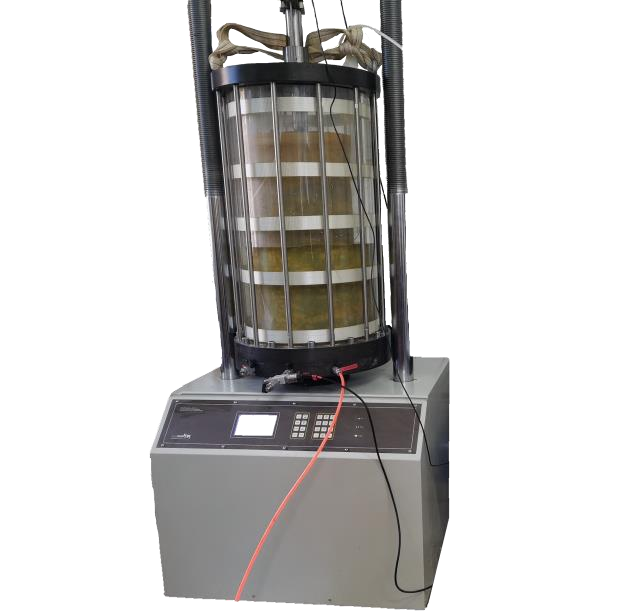


**Fig 3. Figure of the VJT5025‑GDS large‑scale triaxial testing machine**

**Specimen preparation**

The specimens used in this study had a diameter of 300 mm, and the coarse‑grained soil selected for testing contained particles with a maximum size not exceeding 60 mm. The specimens were prepared at their natural water content, and after mixing, the soil was placed in an iron tray and allowed to stand for more than 12 hours to ensure a uniform distribution of moisture.

**Experimental Procedures**

（1） Specimen installation proceeded as follows: an impermeable plate, a latex membrane, and a forming sleeve were first mounted on the base of the pressure chamber. The soil was then placed in layers and compacted to the target density. After compaction, both ends of the latex membrane were secured, and the forming sleeve was subsequently removed.

（2） Assembly and preparation of the pressure chamber proceeded as follows: the specimen was placed inside the chamber with the bottom porous stone aligned with the interface of the pore water pressure sensor, while the top of the specimen was connected to the piston rod to ensure uniform axial loading. The chamber lid was then secured, and the confining pressure line, axial loading system, and sensor cables were connected. The bottom porous stone was linked to the pore water pressure sensor to ensure that the pore water pressure was transmitted through the bottom drainage line to the sensor, and the latex membrane provided an effective seal to prevent chamber water from infiltrating the specimen.

（3） Instrument calibration and data recording were conducted as follows: the lifting platform of the loading frame was adjusted to bring the specimen cap, piston, and load cell into firm contact. The axial deformation gauge was then installed, and the initial measurements were recorded.

（4）Application of pressure proceeded as follows: the drainage valves at the top and bottom of the specimen were closed to maintain undrained conditions during the application of confining pressure. The confining pressure valve was then opened, and the pressure was gradually increased to the predetermined level.

（5） The shearing operation proceeded as follows: the shearing program was initiated, and the shear rate was set to 3 mm/min. The test was stopped when the shear displacement reached 160 mm. During shearing, the deviator stress (*σ*_1_-*σ*_3_) generated an increment in pore water pressure, Δu, and the total pore water pressure was expressed as u = u1 + Δu. The data acquisition system simultaneously recorded u, ε, and (*σ*_1_-*σ*_3_). Although pore water pressure varied with increasing deviator stress during shearing, these data were used solely for process documentation. The final strength parameters were determined based on total stress, without applying any effective‑stress correction.

（6） At the conclusion of the test, axial and confining pressures were first reduced to zero, and the water inside the pressure chamber was drained. The axial pressure and confining pressure were then released in sequence, followed by opening the vent port and drainage valves. After all residual water had been completely drained, the chamber cover was removed. Moisture surrounding the latex membrane was wiped away, the membrane was detached, and the specimen was disassembled.

**Data processing and calculation**

（1）The axial strain was determined using the following equation:

$$\varepsilon_{1}=\frac{\text{Δ}h_{i}}{h_{_{0}}}\times100$$

Where $\varepsilon_{1}$ represents the axial strain (%); denotes the change in specimen height during shearing (mm); $h_{0}$ is the initial height of the specimen (mm).

（2） A relationship curve was constructed with axial strain on the horizontal axis and the principal stress difference on the vertical axis. If a peak was present on the curve, that peak was identified as the failure point; if no distinct peak was observed, the point corresponding to an axial strain of 15% was selected as the failure point.

（3） In a Cartesian coordinate system, the axial stress σ_1_ corresponding to the failure point was plotted on the vertical axis, while the confining pressure σ_3_ was plotted on the horizontal axis for all test data points. The optimal relationship curve was then derived using either a graphical method or the least‑squares method. If the resulting curve was linear, the cohesion and internal friction angle could be directly calculated using the following equations.

Where *c* represents the cohesion of the coarse-grained soil (kPa); $\text{φ}$ denotes the internal friction angle of the coarse-grained soil (°); $\sigma_{c}$ is the intercept of the optimal relationship curve on the vertical axis (kPa); *m* is the slope of the optimal relationship curve.
