# Supplementary material for: Mechanisms of deformation and failure in colluvial slope under artificial surcharge loading
Source: PLoS One. 2026 Jan 5;21(1):e0339772. doi: 10.1371/journal.pone.0339772 (PMC12768376; doi:10.1371/journal.pone.0339772)
Supplement: Attachment — Submitted filename: Response to Reviewers.docx [file pone.0339772.s003.docx]

Dear Editor and Reviewers:

Thank you very much for giving us an opportunity to revise the above manuscript. We appreciate your comments. According to the Editors and Reviewers’ recommendations, we have revised carefully our manuscript. Below are listed our responses to all comments point by point.

Comments from the Editors and Reviewers:

Editor:

1. Please ensure that your manuscript meets PLOS ONE's style requirements, including those for file naming. The PLOS ONE style templates can be found at

https://journals.plos.org/plosone/s/file?id=wjVg/PLOSOne formatting sample main body.pdf and

https://journals.plos.orgLplosone/s/file?id=ba62/PLOSOne formatting sample title authors affiliations.pdf

Thank you for your comments. We have revised the manuscript in accordance with the PLOS ONE template to ensure that it fully meets the required standards.

2. We note that your Data Availability Statement is currently as follows: All relevant data are within the manuscript and its Supporting Information files

Please confirm at this time whether or not your submission contains all raw data required to replicate the results of your study. Authors must share the "minimal data set" for their submission. PLOS defines the minimal data set to consist of the data required to replicate all study findings reported in the article, as well as related metadata and methods

(<https://journals.plos.org/plosone/s/data-availability#loc-minimal-data-set-definition>)

For example, authors should submit the following data:

-The values behind the means, standard deviations and other measures reported;

-The values used to build graphs;

-The points extracted from images for analysis.

Authors do not need to submit their entire data set if only a portion of the data was used in the reported study. If your submission does not contain these data, please either upload them as Supporting Information files or deposit them to a stable, public repository and provide us with the relevant URLs, DOIs, or accession numbers. For a list of recommended repositories, please see https://journals.plos.org/plosone/s/recommended-repositories.

If there are ethical or legal restrictions on sharing a de-identified data set, please explain them in detail (e.g., data contain potentially sensitive information, data are owned by a third-party organization, etc.) and who has imposed them (e.g. an ethics committee).Please also provide contact information for a data access committee, ethics committee, or other institutional body to which data requests may be sent. If data are owned by a third party, please indicate how others may request data access.

Thanks for your comments. The data used in the manuscript will be uploaded as supporting information through the submission system.

3. We notice that your supplementary figures are uploaded with the file type 'Figure' Please amend the file type to 'Supporting Information'. Please ensure that each Supporting Information file has a legend listed in the manuscript after the references list.

Thanks for your comments. We have updated the file types and ensured that each supporting information file contains an appropriate legend placed after the reference list.

4. Please upload a copy of Figures 1-17, to which you refer in your text on pages. If the figures are no longer to be included as part of the submission please remove all reference to it within the text.

Thanks for your comments. A copy of the figure has been submitted as supporting information.

5. Please upload a copy of Supporting Information Tables 1-7 which you refer to in your text on pages 27-28.

Thanks for your comments. We have completed the file upload.

6. If the reviewer comments include a recommendation to cite specific previously published works, please review and evaluate these publications to determine whether they are relevant and should be cited. There is no requirement to cite these works unless the editor has indicated otherwise.

Thanks for your comments. We have completed the review and evaluation.

Reviewer: 1

Comments to the Author

This manuscript Number PONE-D-25-46431( has the potential to be a valuable contribution to the field of engineering geology and slope stability. The core research-integrating physical modeling and numerical analysis to decipher the failure mechanism under external load-is sound and relevant. However, significant revisions are required to address the major concerns regarding methodological clarity, data presentation, and the justification of modeling choices. Once these issues are resolved, the manuscript will be much stronger and suitable for publication.I recommend that the authors carefully address all points raised in this review, particularly the clarification of the model-prototype scaling and the resolution of the data availability statement in line with journal policy.

**Main Comments:**

1. The title of the paper is not informative and not clear, so I suggest to the authors to revise the title.

We sincerely appreciate your valuable suggestion. We have updated the title.

"Title: Mechanisms of Deformation and Failure in Colluvial Slope under artificial surcharge loading"

2. The research contributions of the paper should be articulated more clearly. The abstract is not representative of the content and contributions of the paper. The abstract does not seem to properly convey the rigor of research. And also l recommends the author(s) to be more direct to cover the research gap, manuscript's goal, materials and methods, main results, main contributions, main suggestions for future research.

We sincerely thank you for this helpful observation. We have revised the abstract by more clearly articulating the study’s contributions and presenting a more direct explanation of the research gap, study objectives, materials and methods, key results, and major contributions.

"Abstract: Large-scale artificial loading often triggers landslides in colluvial deposits, yet the mechanical response and failure mechanisms of such slopes under loading remain insufficiently understood. Using a typical colluvial slope in Tibet as a case study, this research integrates physical model tests and FLAC^3D^ simulations to investigate loading-induced deformation and failure processes. Analogous materials composed of river sand, barite powder, calcium carbonate powder, and water were prepared, and multiple regression analysis was used to establish empirical relationships between mix ratios and the resulting cohesion and internal friction angle, yielding high similarity ($R_{a}^{2}$= 0.8732-0.9326). Under loading, the slope exhibits maximum vertical and horizontal displacements of 40 mm and 50 mm, respectively, with shear stress concentrated along the loading boundary and vertical stress penetrating deeper than horizontal stress. The slope undergoes progressive failure: loading → initial equilibrium failure → rear-edge tensile cracking → upper soil mass sliding → front-edge extrusion and bulging → sliding surface propagation → overall failure. Furthermore, the colluvial slope exhibits pronounced failure sensitivity under loading, particularly in the progressive development of rear-edge tensile cracking, toe bulging, and deep shear bands, which should be regarded as key indicators for monitoring. These findings clarify the typical loading-induced failure mechanisms of accumulation-body slopes and provide a scientific basis for early landslide identification and hazard mitigation.

Keyword：Artificial Surcharge Loading; Colluvial Slope; Physical Model Experiment; Slope Deformation and Failure; Similarity Material"

3. The necessity and innovation of the article should be presented to the introduction.

Thank you for this valuable suggestion. We have revised the introduction to articulate more clearly the necessity and originality of the study and to emphasize the distinctions and advancements of this work relative to previous research.

**"Introduction**

......

As an important external load commonly encountered in mountainous transportation engineering, mining operations, and land‑use development [24], surcharge loading is characterized by sudden application, concentrated distribution, and high intensity [25-27]. Such loading can substantially alter the stress and seepage fields of slopes [28], accelerate the accumulation of local damage and the formation of slip surfaces, and consequently cause the failure mode and instability evolution to differ significantly from those under natural conditions [29]. However, existing studies provide insufficient attention to this issue, and systematic investigations into the deformation response, failure‑process evolution, and controlling mechanisms of accumulation‑body slopes under surcharge loading remain limited. This research gap is particularly pronounced in plateau mountainous regions, where large topographic relief and complex rock-soil structures further complicate slope behavior.

To address these gaps, this study investigates a typical deposit slope in Tibet, integrating laboratory physical model experiments with numerical simulations to systematically examine slope deformation and failure characteristics under external loading, while revealing the underlying instability mechanisms. The results provide a theoretical foundation for the siting of major infrastructure and slope-support design, as well as technical guidance for enhancing early detection and risk management of deposit slope failures."

[24] Zhang L, Wang Y, Chen H, Li X, Zhou J, Liu M, et al. Study on slope failure evolution under surcharge loading and toe cutting with BOTDA technology. Opt Fiber Technol. 2021; 66:102644. <https://doi.org/10.1016/j.yofte.2021.102644>.

[25] [Manna B, Biswas S, Roy P, Saha D, Ghosh A, Mukherjee S, et al. Effect of surcharge load on stability of slopes - testing and analysis. Electron J Geotech Eng. 2014; 19:3397-410.](https://www.researchgate.net/publication/289186804_Effect_of_surcharge_load_on_stability_of_slopes_-_testing_and_analysis)

[26] Xu C, Li Y, Zhang H, Chen F, Wang J, Zhou L, et al. Numerical analysis of surcharge effect on stability and interaction mechanism of slope-pile-footing system. J Earth Sci. 2024;35(3):955-69. <https://doi.org/10.1007/s12583-023-1866-7>.

[27] Li N, Cheng YM. Laboratory and 3-D distinct element analysis of the failure mechanism of a slope under external surcharge. Nat Hazards Earth Syst Sci. 2015;15(1):35-43. <https://doi.org/10.5194/nhess-15-35-2015>.

[28] Cheng YM, Li N, Zhang L, Wang H, Chen J, Liu Z, et al. Three-dimensional slope stability problem with a surcharge load. Nat Hazards Earth Syst Sci. 2015;15(10):2227-40. <https://doi.org/10.5194/nhess-15-2227-2015>.

[29] Roy S, Singh A, Kumar P, Sharma R, Patel M, Gupta S, et al. Analysis of unsaturated slope stability under seismic and surcharge loading by upper bound rigid block method. arXiv. 2024; arXiv:2407.16633. <https://doi.org/10.48550/arXiv.2407.16633>.

4. Following, you will find some new related references which should be added to literature review:

- https://doi.org/10.22034/jes.2024.1999733.1029

- https://doi.org/10.1016/B978-0-323-89861-4.00041-5

-	https://doi.org/10.1007/s40996-019-00326-3

Thank you for this valuable suggestion. The literature you provided has been very useful to our work, and we have cited it in the manuscript.

"[1] Arjmandzadeh R, Teshnizi ES, Rastegarnia A, Golian M, Jabbari P, Shamsi H, et al. GIS-based landslide susceptibility mapping in Qazvin Province of Iran. Iran J Sci Technol Trans Civ Eng. 2020;44(S1):S619-S647. <https://doi.org/10.1007/s40996-019-00326-3.>

[59] Hashemi, H., Yazdi, A., & Sharifi Teshnizi, E. Stability analysis of the rock slope of transfer and kinematic analysis: The case study of the Gabric Dam spillway trench head in south of Iran. Journal of Earth & Statistics, 2025; 3(1). <https://doi.org/10.22034/jes.2024.1999733.1029>.

[60] Rahul, S. G., Kumar, N. K., & Kushwaha, R. Smart structural health monitoring system using IoT and wireless technology. In Recent Advancement of IoT Devices in Pollution Control and Health Applications 2023; 85-108. <https://doi.org/10.1016/B978-0-323-95876-9.00008-2.>"

4. For readers to quickly catch your contribution, it would be better to highlight major difficulties and challenges, and your original achievements to overcome them, in a clearer way in abstract and introduction.

Thank you for this constructive comment. We have revised the abstract and introduction to more clearly underscore the primary problems addressed in this study, the challenges encountered, and the original contributions achieved in overcoming these issues.

"**Abstract**:Large-scale artificial loading often triggers landslides in colluvial deposits, yet the mechanical response and failure mechanisms of such slopes under loading remain insufficiently understood. Using a typical colluvial slope in Tibet as a case study, this research integrates physical model tests and FLAC^3D^ simulations to investigate loading-induced deformation and failure processes. Analogous materials composed of river sand, barite powder, calcium carbonate powder, and water were prepared, and multiple regression analysis was used to establish empirical relationships between mix ratios and the resulting cohesion and internal friction angle, yielding high similarity ($R_{a}^{2}$= 0.8732-0.9326). Under loading, the slope exhibits maximum vertical and horizontal displacements of 40 mm and 50 mm, respectively, with shear stress concentrated along the loading boundary and vertical stress penetrating deeper than horizontal stress. The slope undergoes progressive failure: loading → initial equilibrium failure → rear-edge tensile cracking → upper soil mass sliding → front-edge extrusion and bulging → sliding surface propagation → overall failure. Furthermore, the colluvial slope exhibits pronounced failure sensitivity under loading, particularly in the progressive development of rear-edge tensile cracking, toe bulging, and deep shear bands, which should be regarded as key indicators for monitoring. These findings clarify the typical loading-induced failure mechanisms of accumulation-body slopes and provide a scientific basis for early landslide identification and hazard mitigation

**Introduction**

......

As an important external load commonly encountered in mountainous transportation engineering, mining operations, and land‑use development [24], surcharge loading is characterized by sudden application, concentrated distribution, and high intensity [25-27]. Such loading can substantially alter the stress and seepage fields of slopes [28], accelerate the accumulation of local damage and the formation of slip surfaces, and consequently cause the failure mode and instability evolution to differ significantly from those under natural conditions [29]. However, existing studies provide insufficient attention to this issue, and systematic investigations into the deformation response, failure‑process evolution, and controlling mechanisms of accumulation‑body slopes under surcharge loading remain limited. This research gap is particularly pronounced in plateau mountainous regions, where large topographic relief and complex rock-soil structures further complicate slope behavior.

To address these gaps, this study investigates a typical deposit slope in Tibet, integrating laboratory physical model experiments with numerical simulations to systematically examine slope deformation and failure characteristics under external loading, while revealing the underlying instability mechanisms. The results provide a theoretical foundation for the siting of major infrastructure and slope-support design, as well as technical guidance for enhancing early detection and risk management of deposit slope failures."

[24] Zhang L, Wang Y, Chen H, Li X, Zhou J, Liu M, et al. Study on slope failure evolution under surcharge loading and toe cutting with BOTDA technology. Opt Fiber Technol. 2021; 66:102644. <https://doi.org/10.1016/j.yofte.2021.102644>.

[25] [Manna B, Biswas S, Roy P, Saha D, Ghosh A, Mukherjee S, et al. Effect of surcharge load on stability of slopes - testing and analysis. Electron J Geotech Eng. 2014; 19:3397-410.](https://www.researchgate.net/publication/289186804_Effect_of_surcharge_load_on_stability_of_slopes_-_testing_and_analysis)

[26] Xu C, Li Y, Zhang H, Chen F, Wang J, Zhou L, et al. Numerical analysis of surcharge effect on stability and interaction mechanism of slope-pile-footing system. J Earth Sci. 2024;35(3):955-69. <https://doi.org/10.1007/s12583-023-1866-7>.

[27] Li N, Cheng YM. Laboratory and 3-D distinct element analysis of the failure mechanism of a slope under external surcharge. Nat Hazards Earth Syst Sci. 2015;15(1):35-43. <https://doi.org/10.5194/nhess-15-35-2015>.

[28] Cheng YM, Li N, Zhang L, Wang H, Chen J, Liu Z, et al. Three-dimensional slope stability problem with a surcharge load. Nat Hazards Earth Syst Sci. 2015;15(10):2227-40. <https://doi.org/10.5194/nhess-15-2227-2015>.

[29] Roy S, Singh A, Kumar P, Sharma R, Patel M, Gupta S, et al. Analysis of unsaturated slope stability under seismic and surcharge loading by upper bound rigid block method. arXiv. 2024; arXiv:2407.16633. <https://doi.org/10.48550/arXiv.2407.16633>.

6. It is suggested to compare the results of the present research with some similar studies

which is done before.

We sincerely appreciate your valuable suggestion. In Section 5, “Comparative Analysis,” we review and compare slope‑failure mechanisms proposed in relevant literature, including those related to slopes subjected to additional loading and to colluvial slopes under rainfall and seismic loading. The failure mechanisms of colluvial slopes under surcharge loading show notable distinctions. Specifically, the failure behavior of colluvial slopes is jointly governed by the structural characteristics of the colluvial materials, the looseness of joints, and the pronounced stress‑concentration effects induced by localized surcharge loads. Regarding the failure‑evolution process, the behavior exhibits both the “shallow‑to‑deep” shear‑propagation pattern typical of uniformly distributed loads and the directional, wedge‑shaped propagation associated with local loading, which expands “from a point to a plane.” Nevertheless, the final evolution of colluvial landslides generally takes the form of a thrust‑type failure, with the process more strongly influenced by the surcharge location, surcharge width, and the stratified structure of the colluvial deposits. Furthermore, the failure mechanism of colluvial slopes under surcharge loading differs from the traction‑type sliding induced by rainfall and from the thrust‑type instability triggered by earthquakes; instead, it presents a composite failure pattern characterized by cumulative-thrust-structure‑controlled behavior.

**"5.Comparative analysis**

**5.1Studies for comparison with the present work**

To validate the rationality of the deformation and failure mechanism proposed in this study and to highlight the distinct deformation and failure characteristics of accumulation slopes, Table 12 compiles previous research on slope deformation and failure under additional loading for comparative analysis.

**Table 12. Comparison of slope deformation and failure modes under external loading**

| **Source** | **Category** | **Load type** | **Loading position** | **Research method** |
| --- | --- | --- | --- | --- |
| [57] | Homogeneous slope | Uniform load, trapezoidally distributed load and concentrated load | Slope crest | Numerical analysis |
| [58] | Homogeneous slope | Uniform load, embedded load, concentrated load | Slope crest | Numerical analysis combined with experimental testing |

Ref. [57] examined the deformation and failure behavior of slopes subjected to additional loading using numerical analysis. The deformation-failure pattern under a uniform load is illustrated in Fig. 27a. The findings show that, once a uniform load is applied to the slope crest, the slope initially enters an elastic response stage, during which overall deformation remains small and no clear indicators of failure emerge. With continued increases in load magnitude, a shear strain concentration zone develops in the shallow region near the slope crest and gradually evolves into a localized shear band, marking the transition from the elastic stage to the elasto-plastic stage. During this stage, the shallow shear band extends in a strip-like form along the slope surface, representing a characteristic feature of the initial failure process.

As the external load continues to increase, the shallow shear band progressively extends downward and eventually connects to form a continuous weak shear surface, accompanied by a marked enlargement of the plastic zone within the slope. The failure zone follows a characteristic shallow‑to‑deep evolutionary pattern: it initiates with localized damage in the shallow portion of the slope surface, advances gradually into the deeper interior, and ultimately develops into a deep‑seated sliding surface. This progression is also reflected in the displacement-load curve, which exhibits a rapid increase in slope as the load approaches its ultimate value, indicating a significant reduction in slope stiffness and the onset of accelerated instability. Displacement contours from the numerical simulation further demonstrate an overall sliding deformation extending from the slope crest to the toe, with deep‑seated mass movement constituting the dominant failure mode. Failure induced by a uniform load is characterized by a large affected zone, parallel shear‑band development, and a progressively deepening slip surface, highlighting the broad and integral nature of slope failure under uniform loading.

**Fig 27 Failure modes of a homogeneous slope subjected to uniform loading: (a) Failuremode of the slope under a uniform load; (b) Failure mode of the slope under a locally applied uniform load.**

Ref. [58] examined the three‑dimensional slope stability and associated failure mechanisms when a locally applied uniform load was imposed at the slope crest. The deformation and failure pattern under the local uniform load is shown in Fig. 27b. The findings indicate that, unlike the widespread shallow failures induced by a fully uniform load, the slope failure triggered by a local load exhibits pronounced localization and directionality. During the initial loading stage, a distinct stress concentration and the onset of a plastic zone first occur beneath the loaded area, manifested in the rapid development of a localized shear band. The peak shear strain within this zone is substantially higher than that in the surrounding soil, causing the local structural integrity of the slope to be compromised first.

As the load continues to act and the plastic zone expands, the shear band progresses into the slope interior and downward in a wedge‑shaped or tongue‑shaped pattern, exhibiting pronounced three‑dimensional propagation characteristics. Because the loaded area is limited, the failure no longer develops uniformly along the slope surface but instead evolves into an asymmetric shear‑failure path aligned with the direction of stress concentration. The local load also tends to induce tensile cracking at the slope crest. These cracks widen as deformation increases and gradually propagate toward the slope surface, with their trajectory consistent with the contour of the internal shear band, further confirming the localized and directional nature of the failure.

When the localized shear zone expands to a critical scale, the plastic zone extends toward the slope toe and becomes fully connected, forming a composite shear‑sliding surface and marking the onset of overall slope instability. At this stage, the displacement curves show a rapid increase in the displacement difference between the upper and lower segments of the sliding surface, indicating that the sliding mass is transitioning into an accelerated downslope movement phase.

Compared with a uniformly distributed load, failure induced by a local load is more strongly governed by stress‑concentration points. Although the affected zone is relatively limited, the resulting damage is more intense, making rapid localized failure more likely and allowing it to propagate progressively to the entire slope. Overall, under a locally applied uniform load, slope failure exhibits features such as point‑to‑surface evolution, pronounced spatial directionality, and three‑dimensional wedge‑shaped expansion of the shear band, demonstrating the strong perturbation effect of local loading on slope stability.

**5.2 Comparative analysis results**

**5.2.1 Comparison of Failure Mechanisms in Homogeneous Slopes under Additional Loading**

This study compares the slope failure patterns identified herein with those reported in previous studies under uniformly distributed loads and locally applied uniformly distributed loads. The results show that slopes composed of colluvial or accumulative deposits exhibit a characteristic “pushing‑type” failure mode when subjected to surcharge‑induced disturbances. These failure features arise not only from the inherent properties of the deposits—namely weak cementation and low shear strength—but also from the pronounced influence of load type, stress‑transfer pathways, and modes of spatial propagation.

At the initial stage of failure evolution, the slope was primarily undergoing stress redistribution, characterized by the emergence of shallow short cracks, the formation of tensile cracks at the rear edge of the surcharge, and localized settlement near the slope toe. The characteristics of this stage align with the early deformation patterns reported under uniformly distributed loading in Ref. [57], which similarly show that shallow stress perturbations develop first. However, unlike the planar and uniformly applied disturbance produced by a uniformly distributed load, the surcharge in this study functions as an approximately “locally concentrated load.” Consequently, the rear‑edge tensile cracks are more pronounced and display distinct structural‑control features, more closely matching the initiation behavior of localized shear zones induced by locally applied uniformly distributed loads as described in Ref. [58].

As the surcharge continues to act, the slope transitions into a stage of intensified deformation. The number and width of cracks increase markedly, and the crack network expands both inward and laterally within the slope. Penetrating microcracks develop within the interior and gradually evolve into a potential slip surface. This process is consistent with the “shallow-intermediate-deep progressive extension” failure pathway reported under uniformly distributed loading, reflecting the gradual formation of the slip surface. However, the colluvial slope simultaneously exhibits a pronounced coupled response characterized by frontal bulging and rear‑edge downward displacement. The relative movement of the sliding mass becomes more evident, representing a typical pushing‑type structural failure. In contrast, shear bands formed under uniformly distributed loads tend to be more parallel and widespread, whereas those generated by localized loading display stronger directionality and wedge‑shaped propagation. Therefore, the failure mode observed in this study falls between the global extension produced by uniformly distributed loads and the directional extension induced by localized loads, but it more closely resembles failure patterns triggered by localized loading.

At the final failure stage, all three types of slopes exhibit a fully developed slip surface and overall instability of the sliding mass. Under uniformly distributed loading, the slip surface penetrates deeply and continuously, producing large‑scale downslope displacement. Under localized loading, the shear surface typically shows three‑dimensional wedge‑shaped or tongue‑shaped propagation. The pushing‑type failure identified in this study is characterized by a distinct rear scarp, a bulging shear‑out zone at the slope front, and a through‑going slip surface that develops from top to bottom, ultimately forming an integrated sliding accumulation mass. Although this failure mode aligns more closely with the directional failure induced by localized loading, its progressive evolution is more complex, exhibiting a multi‑stage coupled sequence: initiation of rear‑edge tensile cracking → sliding of the upper‑middle block → frontal shear‑bulging extrusion → penetration of the slip surface → overall sliding.

Overall, the failure mechanism of colluvial slopes is jointly governed by the structural characteristics of the colluvial material, the looseness of joints, and the strong stress‑concentration effects induced by localized surcharge loading. In terms of failure evolution, the slopes exhibit both the “shallow‑to‑deep” shear‑extension pattern typical of uniformly distributed loading and the “point‑to‑surface” wedge‑shaped directional propagation associated with localized loading. However, the final evolution of colluvial landslides tends to develop into a typical pushing‑type failure, in which the failure process is more strongly influenced by the surcharge location, surcharge width, and the stratification of the colluvial deposits.

Therefore, the failure mechanism of colluvial slopes under surcharge loading cannot be simply categorized as either the uniformly distributed loading-induced mode or the localized loading-induced mode. Rather, it should be understood as a surcharge‑induced cumulative, pushing‑type, and structurally controlled failure mechanism that develops on the basis of the inherent structural weakness of the colluvial deposits.

**5.2.2 Comparison of Failure Mechanisms in Colluvial Slopes under Rainfall and Seismic Loading**

For the rainfall‑induced failure of colluvial deposits, the comparative reference used in this study is Ref. [16]. Khan et al. investigated the failure mechanism of unsaturated coal‑gangue colluvial slopes subjected to rainfall infiltration [16]. The failure process induced by rainfall infiltration in coal‑gangue slopes evolves through two distinct stages. The first stage is “seepage‑driven saturation and suction loss,” during which rainwater rapidly infiltrates through the highly permeable gangue material, causing matric suction to decrease sharply as the water content increases. Due to its highest permeability, the slope crest becomes saturated first, leading to nonuniform swelling and the formation of tensile cracks. The second stage is “strength degradation and initiation of sliding.” As seepage continues, pore‑water pressure shifts from negative to positive, effective stress progressively decreases, and the shear strength along the potential slip zone significantly deteriorates. Under the combined effects of self‑weight and seepage forces, a traction‑type sliding initiates from the crest and propagates downslope, ultimately reducing the factor of safety to below 0.9 and resulting in global slope failure.

For earthquake‑induced failures in colluvial deposits, Ref. [23] is used as the comparative source in this study. Ma et al. examined the failure mechanisms of colluvial slopes through shaking‑table experiments. The seismic failure process of colluvial‑layer landslides can be summarized in two stages. The first stage is “crack initiation and propagation.” As seismic waves travel upward from the base of the slip zone, the combined effects of slip‑zone strength softening and topographic amplification lead to the formation of tensile cracks in the mid‑slope region. These cracks then propagate toward the slope toe, outlining the initial geometry of a throughgoing slip surface. The second stage is “slip‑surface formation and instability onset.” With continued shaking, the soil within the slip zone undergoes plastic yielding, the point safety factor decreases overall and exhibits a delayed peak, and a deep tensile crack forms at the rear of the sliding mass. The sliding mass subsequently undergoes a pushing‑type displacement from the mid‑slope toward the slope toe, ultimately resulting in global failure as the shear resistance along the slip zone drops sharply.

The failure mechanisms of colluvial slopes under different external disturbances exhibit both distinct differences and shared characteristics. Under rainfall conditions, landslide failure progresses through two stages: rainwater first infiltrates rapidly through highly permeable zones, causing early saturation at the slope crest and the development of tensile cracks; subsequently, rising pore‑water pressure leads to strength degradation along the slip zone, inducing traction‑type sliding from the crest toward the toe. Under seismic loading, failure initiates with tensile cracking in the mid‑slope region, after which the slip zone undergoes plastic yielding, and the sliding mass experiences a pushing‑type displacement from the mid‑slope toward the slope front. In contrast, slope failure induced by surcharge loading in this study exhibits a typical “pushing‑type” evolution. Surcharge‑driven stress redistribution leads sequentially to rear‑edge tensile cracking, crest sliding, and frontal bulging, during which the potential slip surface progressively develops and ultimately forms a tongue‑shaped sliding mass.

From a mechanistic perspective, rainfall‑induced landslides are primarily controlled by increases in pore‑water pressure and the degradation of shear strength, whereas earthquake‑induced landslides are driven by instantaneous shearing and plastic yielding. In contrast, surcharge‑induced landslides are triggered by stress accumulation and localized stress concentration, exhibiting progressive failure with pronounced structural‑control features. The failure process under surcharge loading involves both the “shallow‑to‑deep” shear propagation characteristic of uniformly distributed loads and the wedge‑shaped “point‑to‑surface” propagation induced by localized loads, while more prominently displaying a multistage interaction consisting of rear‑edge tensile cracking, translational sliding of the upper slope, and frontal bulging. Therefore, the failure mechanism of colluvial slopes subjected to surcharge loading is unique: it differs from the traction‑type sliding induced by rainfall and from the pushing‑type instability caused by earthquakes, instead manifesting as a cumulative-pushing-structurally controlled composite failure mode.

**5.2.3 Failure Mechanisms in Comparison with Rock Slopes**

In addition, colluvial slopes differ markedly from rock slopes, with their failure patterns more strongly governed by material weakness and structural looseness. Because rock masses possess higher structural integrity and more regular joint-fracture networks, the failure of rock slopes is typically controlled by geometrically well‑defined combinations of structural planes, resulting in structural instability modes such as block sliding, wedge failure, or toppling, with the failure surface being relatively predictable [59]. In contrast, colluvial slopes consist of rock fragments, soil materials, and loose accumulations, and their internal structure is highly heterogeneous. Their deformation generally develops through progressive plastic propagation, and their sliding surfaces tend to be more tortuous, less continuous, and more susceptible to local disturbances. Compared with the “structure‑controlled overall sliding” commonly observed in rock‑slope failures, colluvial slopes more readily initiate microcracks at sites of local stress concentration and progressively evolve along weak, poorly persistent zones under sustained surcharge loading, ultimately forming a pushing‑type overall slide.

Meanwhile, the differing responses of rock slopes under uniformly distributed loading and local loading are mainly reflected in the extent to which structural planes participate in deformation and in the geometric variations of shear bands. In contrast, because particle contacts within colluvial slopes are inherently loose, local surcharge loading more readily induces pronounced stress‑concentration effects, resulting in coupled deformation characterized by rear‑edge tension cracking, front‑edge bulging, and segmented displacement of the sliding mass. This leads to an overall evolutionary pattern of “zonal response-coupled failure” [59].

In summary, from an engineering perspective, colluvial slopes show pronounced failure sensitivity under surcharge loading. The progressive development of rear‑edge tension cracking, front‑edge bulging, and deep shear bands should be regarded as key monitoring indicators. Controlling surcharge intensity, limiting the spatial extent of surcharge loading, mitigating stress concentration at the surcharge-slope interface, and improving the shear strength of both shallow and deep layers of the colluvial deposits are essential measures for preventing surcharge‑induced landslides. In landslide‑prone regions, dynamic monitoring systems can be employed for continuous observation [60]."

[57] Leeworthy, T., & Ahangar Asr, A. Effects of various surcharge loading conditions on the stability of soil slopes. Paper presented at the UK Association for Computational Mechanics 2020. https://doi.org/10.17028/rd.lboro.12097698.v1.

[58] Cheng, Y. M., Li, N., & Yang, X. Q. Three-dimensional slope stability problem with a surcharge load. Natural Hazards and Earth System Sciences, 2015; 15(10). https://doi.org/10.5194/nhess-15-2227-2015.

[59] Hashemi, H., Yazdi, A., & Sharifi Teshnizi, E. Stability analysis of the rock slope of transfer and kinematic analysis: The case study of the Gabric Dam spillway trench head in south of Iran. Journal of Earth & Statistics, 2025; 3(1). https://doi.org/10.22034/jes.2024.1999733.1029.

[60] Rahul, S. G., Kumar, N. K., & Kushwaha, R. Smart structural health monitoring system using IoT and wireless technology. In Recent Advancement of IoT Devices in Pollution Control and Health Applications 2023; 85-108. https://doi.org/10.1016/B978-0-323-95876-9.00008-2.

7. Much more explanations and interpretations must be added for the Results, that's not

enough.

We sincerely thank you for this helpful observation. In the Results section, we provide additional explanations and clarifications. Section 3.2.2 now includes an expanded analysis of the plastic zone, and Section 4.2 presents the overall deformation‑and‑failure process of the colluvial slope, accompanied by a quantitative assessment.

"**3.2.3 Plastic Zone Analysis**

In the numerical analysis, a detailed examination of the plastic zones enables effective identification of the deformation characteristics of the slope body and provides insight into the stress state of individual slope elements.This analysis allows a clear distinction between tensile and shear failure regions within the slope.Fig 25 presents the distribution of plastic zones under different working conditions.

**Fig. 25 Horizontal displacement contours of the slope model under different conditions: (a) distance 10 m, load 200 kPa; (b) distance 20 m, load 200 kPa; (c) distance 10 m, load 300 kPa; (d) distance 20 m, load 300 kPa; (e) distance 10 m, load 400 kPa; (f) distance 20 m, load 400 kPa**

According to the figure, tensile plastic zones appear at both the front and rear edges of the surcharge area at the slope crest, indicating that the soil in this region primarily undergoes tensile failure. Under the same surcharge, shear plastic zones develop within the surcharge area, in the underlying slope body, and at the slope toe. This phenomenon indicates that the soil in these regions fails predominantly through shear deformation, meaning that the soil beneath the surcharge area and at the slope toe gradually experiences failure due to shear forces induced by the surcharge.

Furthermore, due to the stress transfer and redistribution induced by the surcharge, the shear stress acting on the soil near the surcharge area gradually increases. Once this stress exceeds the soil’s shear strength, a shear failure zone forms and progressively extends toward the middle of the slope as the surcharge persists or related influencing factors continue. Under the combined effects of the surcharge and the self‑weight of the slope, the slope toe is subjected to elevated shear stress. When this stress surpasses the soil’s shear strength, shear failure initiates at the slope toe and subsequently propagates upslope toward the slope’s central region. Consequently, shear failure zones originating beneath the surcharge area and at the slope toe expand progressively, resembling two extending “failure bands” that advance toward the middle of the slope. Over time, these bands eventually intersect and merge, further intensifying soil failure and posing a significant threat to the overall stability of the slope.

**4.2 Embankment Slope Deformation and Failure under Surcharge Loading**

Based on physical model experiments and numerical analysis, this study summarizes the failure mechanism of deposit slopes under applied loading, which can be divided into three main stages, as shown in Fig 26.

**Fig. 26 Three stages of deformation and failure in an accumulation slope**

......

As time progressed and the surcharge load continued to increase, the maximum earth pressure at Monitoring Point No. 4 near the slope crest reached 1.88 kPa, while that at Monitoring Point No. 5 near the slope toe reached 1.85 kPa, exceeding the soil’s yield strength and triggering slope deformation and failure. The failure was mainly characterized by progressive crack propagation and penetration, accompanied by the formation of an internal slip surface. Uplift occurred at the slope toe within the surcharge zone, and Displacement Gauge No. 3 recorded a maximum horizontal displacement of 12 mm, indicating substantial lateral thrust that induced shear failure and produced a tongue‑shaped accumulation body. According to the displacement data, the maximum horizontal displacements at Points A3 (upper part of the slip belt), A2 (middle part), and A1 (lower part) were 7 mm, 50 mm, and 18 mm, respectively. A scarp and steep face developed at the rear of the surcharge zone, signifying a complete loss of the slope’s bearing capacity. Overall, the failure mechanism involved stress concentration that initiated and propagated cracks, the formation of a potential slip surface, and ultimately toe bulging, shearing, and overall sliding failure.In summary, the formation and evolution of slope failure under applied loading follows a “progressive migration” mechanism: loading → initial equilibrium disruption → rear-edge tensile cracking → upper soil sliding → front-edge compression and bulging → sliding surface propagation and interconnection → overall slope sliding failure."

8. Some key parameters are not mentioned. The rationale on the choice of the particular set of parameters should be explained with more details. Have the authors experimented with other sets of values? What are the sensitivities of these parameters on the results?

Thank you for this valuable suggestion. In Section 2.1.1 of the methodology, we present a detailed rationale for the selection of parameters, and in Section 2.1.2, we further examine how mix‑proportion factors affect these parameters.

"**2.1.1 Similarity Material Proportioning**

......Studies have shown that the shear strength parameters of geotechnical materials—cohesion c and internal friction angle $\varphi$ are key factors governing the slope safety factor. These parameters directly influence both the occurrence of slope failure and the potential failure mode, and thus should be treated as primary variables during material proportioning [51-54].

**2.1.2 Influence Mechanisms of Various Factors on Similar Materials**

This study employed the range analysis method to examine how the four component factors A, B, C, and D influence the material’s physical-mechanical parameters and hydraulic properties.The range is calculated as the difference between the maximum and minimum average values across the levels of each factor.A larger range indicates that the corresponding factor exerts a stronger influence on the properties of the similar materials.

**2.1.2.1 Analysis of Factors Affecting the Density of Similar Materials**

Based on the orthogonal test results, a range analysis was performed to evaluate the density of the similar materials.The average values for each level of factors A-C were calculated, and the corresponding line chart was plotted, as shown in Fig 8.The results indicate that the relative influence of the factors on the density of the similar materials decreases in the order of B (range: 0.077, with subsequent values also representing ranges)> A (0.0575)> and C (0.0503).Factor B (barite powder/aggregate) is the primary factor controlling the density.A lower barite powder content combined with higher contents of river sand and heavy calcium powder leads to an increase in the density of the similar materials.This finding further confirms that incorporating river sand and heavy calcium powder into the aggregate can effectively regulate the unit weight of similar materials

**Fig. 8 Analysis of density sensitivity factors**

**2.1.2.2 Analysis of Factors Affecting the Cohesion and Internal Friction Angle of Similar Materials**

Based on the test results for each group of similar materials, the average cohesion and internal friction angle corresponding to each factor were calculated under both natural and water‑saturated conditions, and the resulting line chart is presented in Fig 9.

**Fig. 9 Analysis of sensitivity factors affecting cohesion and internal friction angle**

Based on the calculations, the influence of each factor on cohesion is ranked as A (0.405) > C (0.350) > B (0.330).This result indicates that the sensitivity of cohesion is primarily governed by factor A (river sand/aggregate).As the river sand content increases, the interlocking and contact characteristics among the particles of the similar materials are altered, promoting the formation of a more stable internal structural framework that enhances cohesion.Although double fly ash powder and barite powder also exert an influence on cohesion, their effects are weaker compared with the changes induced by variations in aggregate particle gradation.These findings suggest that the cohesion of similar materials is predominantly controlled by the configuration of the aggregate skeleton, while fine‑grained materials mainly serve as fillers and provide localized cementation.

Similarly, the influence of each factor on the internal friction angle is ranked as B (2.14) > C (1.07) > A (0.74).Among these factors, factor B (barite powder/aggregate) exerts the most significant and dominant control over the internal friction angle.Variations in barite powder content lead to substantial changes in particle morphology, compactness, and interparticle friction within the similar materials, resulting in pronounced differences in their frictional behavior.Barite powder is characterized by its high density and relatively smooth, low‑angularity particles; therefore, when its content decreases, the rougher particle surfaces formed by river sand and double fly ash powder, together with increased aggregate contact strength, contribute to an increase in the internal friction angle.It is also notable that factor C (double fly ash powder/aggregate) has a measurable influence on the internal friction angle, primarily because double fly ash powder fills the voids within the skeleton and adjusts particle packing, thereby indirectly modifying the material’s overall shear‑resistance system.

In summary, cohesion is primarily governed by the structural characteristics of the aggregate particle framework, whereas the internal friction angle is mainly controlled by interparticle frictional resistance and material compactness.The differing sensitivities of these two strength parameters to the various factors reflect the distinct dominant mechanisms associated with internal structure and particle gradation during the shear behavior of similar materials.These results provide a reliable basis for optimizing mixture proportions and conducting subsequent model tests involving similar materials.

**2.1.3 Multivariate Regression Analysis of Parameters and Determination of Experimental Material Mix Proportions**

In this study, multivariate regression analysis was conducted to quantify the relationships between each physical and mechanical parameter and the compositional proportions of the three factors A, B, and C.The fitting results for each parameter are presented in Fig 10.The coefficients of determination between the regression‑derived fitted values and the corresponding experimental values range from $R_{a}^{2}$ = 0.8732 to 0.9326, indicating a strong goodness of fit.The regression equations are provided in Eq 5.

(5)

In the equation, a represents the percentage of river sand mass relative to the aggregate mass; b represents the percentage of barite powder mass relative to the aggregate mass; and c represents the percentage of double‑fly ash powder mass relative to the aggregate mass.

**Fig. 10 Comparison of the multivariate regression fitting results with the experimental results for each parameter**

[51] Wang M, Li L. Slope Stability Analysis Considering Degradation of Soil Properties Induced by Intermittent Rainfall. Water. 2025; 17(6):814. https://doi.org/10.3390/w17060814

[52] He, Y., Li, Z., Wang, W. et al. Slope stability analysis considering the strength anisotropy of c-φ soil. Sci Rep 12, 18372 (2022). https://doi.org/10.1038/s41598-022-20819-y

[53] Nassirzadeh, R., Dini, A. & Balagar, V. Back analysis of cohesion and friction angle of failed slopes using probabilistic approach: two case studies. Geo-Engineering 15, 3 (2024). https://doi.org/10.1186/s40703-024-00205-5.

[54] Harabinová, S., & Panulinová, E. (2020). Impact of shear strength parameters on slope stability. MATEC Web of Conferences, 310, 00040. https://doi.org/10.1051/matecconf/202031000040"

9. You should add the flow chart in this manuscript for methodology.

Thank you for this constructive comment. We have included an overall methodological flowchart in Section 2.

"The primary research methods used in this study are laboratory model testing and numerical simulation. First, an indoor physical model test was conducted by preparing similar materials based on data obtained from field investigations and geological surveys. In view of the inherent limitations of physical model testing, numerical simulations were subsequently performed according to similarity relationships, using the prototype dimensions along with the corresponding load levels and loading durations. Integrating these two approaches enables both comparative verification and a deeper interpretation of the deformation and failure mechanisms of accumulation‑body slopes under surcharge loading. The overall workflow is illustrated in Fig. 3"

**Fig. 3 Overall workflow of the research methodology"**

10. The conclusion section would be improved by starting with the research goal. The author could present the practical implication of this study. However, further suggestions for future research could be addressed and inserting a paragraph by stating the limitations of this study.

Thank you for this valuable suggestion. We have revised the conclusion section by restating the research objectives, clarifying the study’s practical significance, offering recommendations for future research, and adding a paragraph that outlines the limitations of this study.

"**6.Conclusion**

This study aims to systematically elucidate the deformation‑response characteristics and failure mechanisms of colluvial slopes subjected to surcharge loading. By integrating physical model tests with FLAC3D numerical simulations, the study provides a comprehensive investigation of surcharge load transfer, stress distribution, deformation paths, and failure‑process evolution. Furthermore, a multivariate regression‑based similarity‑material proportioning method is proposed, offering systematic theoretical and methodological support for stability analyses of colluvial slopes under artificial loading. The main conclusions are as follows:

(1)Under surcharge loading, the maximum vertical displacement of 40 mm occurs at the slope crest, while the maximum horizontal displacement of 50 mm develops in the steeper mid‑slope region. The influence depth of vertical stress is substantially greater than that of horizontal stress, and the maximum shear stress is concentrated along the edge of the surcharge zone. The vertical stress beneath the surcharge area exhibits a depth‑dependent attenuation pattern.

(2) As the surcharge load increases, both the extent and intensity of regions exhibiting large shear‑strain increments expand, and the shear band progressively propagates from the slope crest toward the slope toe. The shear‑failure zones that form within the slope beneath the edge of the surcharge area and at the slope toe tend to extend toward the mid‑slope region.

(3) Under the same surcharge load, zones exhibiting pronounced shear‑strain increments are more likely to form closer to the slope‑break line. At a given surcharge location, a larger load leads to a greater extent of shear‑strain‑increment zone propagation toward the slope toe.

(4) The failure mode of accumulation‑slope masses under surcharge loading differs from that of homogeneous‑slope landslides and those induced by other loading conditions. The formation and evolutionary mechanism of surcharge‑triggered landslides proceeds as follows: surcharge application → disturbance of the initial equilibrium → initiation of rear‑edge tensile cracking → sliding of the upper soil mass → frontal compression and bulging → propagation and coalescence of the sliding surface → overall sliding failure.

(5) Accumulation slopes exhibit marked failure sensitivity under surcharge loading, and the progressive development of rear‑edge tensile cracking, frontal bulging, and deep‑seated shear zones should be regarded as key indicators for monitoring.

Due to limitations in the experimental conditions, this study still has several constraints:
(1) The experiment adopted only a static, staged surcharge‑loading scheme and did not consider influences such as rapid load application during construction, mechanical vibration, or traffic‑induced dynamic loading.(2) To simplify the computations, the numerical model employed the Mohr-Coulomb constitutive relation and did not incorporate mesoscopic effects such as structural‑plane weakening or particle breakage, which may cause the simulation results to be somewhat conservative or idealized.

Future research can be further advanced in the following directions:(1) Incorporating high‑precision in situ monitoring data to establish a multi‑scale validation framework that integrates experimental, numerical, and field observations.(2) Investigating mesoscopic failure mechanisms using discrete‑element or particle‑flow methods to clarify the fundamental influences of particle gradation and structural characteristics.(3) Introducing dynamic loading and unloading effects to simulate surcharge‑induced disturbance responses under realistic construction conditions.(4) Conducting multi‑field coupling studies involving surcharge loading, rainfall, seepage, and seismic actions to develop a comprehensive stability‑assessment framework for more complex environmental settings.

Major Points of Concern

1. Clarity and Coherence of the Experimental and Numerical Setup:

1) Scale and Units: There is a critical and confusing discrepancy in the scaling and units between the physical model and the numerical simulation. The physical model uses a geometric scale of 1:200 and applies a maximum load of 2.0 kPa. The numerical simulation, intended to represent the prototype, applies loads of 200-400 kPa. The relationship between these loads is not explained. ls the 2 kPa a model-scale load? If so, what is the corresponding prototype load (it should be 400 kPa, given the geometric scale)? This fundamental relationship must be explicitly stated and justified.

Thank you for this valuable suggestion. First, we supplemented the derivation of the similarity parameters in Section 2.1.1 of the manuscript and presented the similarity ratios in Table 1. Because the actual loads acting on the phase‑field slope were unavailable and considering the inherent limitations of the physical model test, the loads applied in the numerical simulation were converted from the physical model test loads according to the similarity ratios, while the geometric dimensions were based on the field scale. Consequently, following the similarity relationships, the maximum load in the physical model was 2  kPa, whereas the maximum load in the numerical simulation was 400  kPa, as also explained in Section 2.2.2.

"**2.1.1 Similarity Material Proportioning**

The development of analogous materials is a prerequisite for conducting physical model experiments and is a critical factor in determining whether the model can accurately replicate the physical properties of the prototype [32]. Based on similarity theory, this study establishes quantitative relationships between the model and the prototype to reveal the underlying mechanisms of complex phenomena. To ensure that the model’s deformation behavior corresponds to that of the actual engineering system, the model must satisfy the required similarity criteria. All relevant parameters can be represented by the following relationship, as shown in Eq 1.

(1)

In Eq 1, l denotes the model size, γ is the unit weight of the model, g represents gravitational acceleration, c is cohesion, φ is the internal friction angle, E is the elastic modulus of the model, δ is displacement, σ is stress, ε is strain, μ is Poisson’s ratio, t is time, and F is force. Because φ, ε, and μ are dimensionless quantities, their similarity ratios are equal to 1, that is, Cφ = Cμ = Cε = 1. When Cγ is set to 1, the derivation of the remaining physical quantities must satisfy the similarity criteria specified in Eqs 2-4.

$C_{l}=l_{p}/l_{m}$ (2)

In the equation, Cl is the geometric similarity constant with the dimension of length, where the subscript p denotes the prototype and the subscript m denotes the model.

$\left\{\begin{matrix} C_{\sigma}=C_{l}C_{\gamma} \\ C_{\sigma}=C_{E}C_{\varepsilon} \\ C_{\delta}=C_{l} \\ C_{F}=C_{\sigma}C_{l}^{2} \end{matrix}\right.$ (3)

In the equation, Cσ denotes the stress similarity constant, Cγ represents the unit‑weight similarity constant, CE is the elastic modulus similarity constant, Cε is the strain similarity constant, Cδ is the displacement similarity constant, and CF is the force similarity constant.

$\left\{\begin{matrix} C_{\sigma}=C_{R}^{\tau} \\ C_{c}=C_{R}^{\tau} \\ C_{R}^{c}=C_{R}^{\tau} \end{matrix}\right.$ (4)

In the equation, $C_{R}^{\tau}$ denotes the tensile strength similarity constant, $C_{c}$ represents the cohesion similarity constant, and $C_{R}^{c}$ is the compressive strength similarity constant.

The principal geometric dimensions of the debris‑slope cross‑section are shown in Fig 5. To ensure experimental operability, the geometric similarity constant was set to 200 in the model tests, and the similarity ratios for the other physical quantities are provided in Table 1.

**Fig. 5 Geometric dimensions of the debris‑slope cross‑section**

**Table 1. Similarity parameters between model and prototype**

| **Physical Quantity** | **Dimension** | **Similar Quantity** | **Similarity Ratio** |
| --- | --- | --- | --- |
| Geometric length (*L)* | L | $C_{L}$ | 200 |
| Internal friction ($\varphi$) | 1 | $C_{\varphi}$ | 1 |
| Cohesion (*c*) | FL^-1^T^-2^ | $C_{c}$ | 200 |
| Water content (*ω*) | 1 | $C_{\omega}$ | 1 |
| Unit weight (*γ*) | FL^-3^ | *C_γ_* | 1 |
| Strain (*ε*) | 1 | *C_ε_* | 1 |
| Elastic modulus (*E*) | FL^-2^ | *C_E_* | 200 |
| Poisson’s ratio (*μ*) | 1 | C*_μ_* | 1 |
| Displacement (*δ*) | L | C*_δ_* | 200 |
| Stress (*σ*) | FL^-2^ | C*_σ_* | 200 |
| Force (*F*) | F | *C_F_* | 200^3^ |
| Flexural rigidity (*EI*) | FL^2^ | C*_EI_* | 200^5^ |
| Time (*t*) | T | *C_t_* | 200^1/2^ |

**2.2.2 Determination of Soil Parameters through Inversion and Load Parameter Specification**

For the computational conditions, the numerical model was assumed to consist of a homogeneous soil mass, and the simulations primarily considered the load magnitude and its position at the slope crest.The surcharge area was defined using geometric coordinates or local mesh refinement, after which a uniformly distributed surface load was applied to generate vertical stress, ensuring that the load acted solely on the ground surface without propagating into deeper layers.Based on the surcharge width used in the physical model tests, the surcharge area was set to a width of 20 m.Six surcharge combinations were designed, as summarized in Table 11.

**Table 11. Loading combinations for the slope in the numerical simulations**

| **Distance from slope crest(m)** | **Applied load(kPa)** | | |
| --- | --- | --- | --- |
| 10 | 200 | 300 | 400 |
| 20 | 200 | 300 | 400 |

2) Numerical Model Justification: The manuscript states that numerical simulation is used to overcome the limitations of the physical model (lines 235-242). However, it then uses a" homogeneous soil mass" for the simulation, whereas the physical model and the field site are clearly composed of multiple strata (Q4del, Q4al+pl, Q3fal). This simplification undermines the goal of validating the experimental results and representing the prototype. The authors must justify why a homogeneous model was chosen and discuss how this affects the comparability of the results.

Thanks for your comments. We apologize for the earlier misexpression. In our manuscript, the term “homogeneous soil” was intended to indicate that the soil properties within each layer are uniform. We have revised the wording accordingly.

"**2.2.2 Determination of Soil Parameters through Inversion and Load Parameter Specification**

For the computational conditions, the numerical model was assumed to consist of a homogeneous soil mass, and the simulations primarily considered the load magnitude and its position at the slope crest.The surcharge area was defined using geometric coordinates or local mesh refinement, after which a uniformly distributed surface load was applied to generate vertical stress, ensuring that the load acted solely on the ground surface without propagating into deeper layers.Based on the surcharge width used in the physical model tests, the surcharge area was set to a width of 20 m.Six surcharge combinations were designed, as summarized in Table 11."

**Table 11. Loading combinations for the slope in the numerical simulations**

| **Distance from slope crest(m)** | **Applied load(kPa)** | | |
| --- | --- | --- | --- |
| 10 | 200 | 300 | 400 |
| 20 | 200 | 300 | 400 |

2. Data Availability Statement:

The journal's policy explicitly states that " 'data available on request from the author' is not sufficient." The current Data Availability Statement (line 471) contradicts this policy. The authors must either:

1) Deposit all relevant data in a public repository (e.g. figshare, Zenodo ) and provide the accession link/DOI.

2) State unequivocally that all data are within the manuscript and its Supporting Information files (which currently do not seem to include the raw data, only figures).

3) Provide a compelling, journal-compliant reason for restricting data access.

We sincerely apologize for the limitations imposed by relevant regulations in China. According to the data confidentiality requirements of national laboratories, only test data that have undergone open review may be disclosed. Furthermore, based on the conditions of the field investigation and the requirements of the local governmental authorities, the original site exploration data cannot be released in detail. Therefore, we have provided the relevant figures and supplementary supporting information, and we kindly request your understanding.

3. Presentation and Interpretation of Results:

1) Figure Referencing: All figures are cited as "S1 Fig,"S2 Fig," etc. in the text, but the corresponding captions and images are embedded at the end of the PDF without formal captions. This makes it extremely difficult to follow the narrative. The figures themselves(e.g., S16-Fig.16.tif) are also very small and lack clear, readable labels and scales. The figures must be properly formatted with high resolution and descriptive captions.

Thanks for your comments. We have revised the relevant figures and added captions accordingly.

2) Quantitative Analysis: The results are largely descriptive. A more quantitative analysis would significantly strengthen the paper. For instance:

①The failure process described in lines 432-437 is a key finding. This should be directly linked to quantitative thresholds from the model (e.g., "rear-edge tensile cracking initiated when the load reached X kPa and the horizontal displacement at sensor X exceeded Ymm").

Thanks for your comments. We have made the corresponding revisions in the relevant sections.

"**4.2 Embankment Slope Deformation and Failure under Surcharge Loading**

......

As time progressed and the surcharge load continued to increase, the maximum earth pressure at Monitoring Point No. 4 near the slope crest reached 1.88 kPa, while that at Monitoring Point No. 5 near the slope toe reached 1.85 kPa, exceeding the soil’s yield strength and triggering slope deformation and failure. The failure was mainly characterized by progressive crack propagation and penetration, accompanied by the formation of an internal slip surface. Uplift occurred at the slope toe within the surcharge zone, and Displacement Gauge No. 3 recorded a maximum horizontal displacement of 12 mm, indicating substantial lateral thrust that induced shear failure and produced a tongue‑shaped accumulation body. According to the displacement data, the maximum horizontal displacements at Points A3 (upper part of the slip belt), A2 (middle part), and A1 (lower part) were 7 mm, 50 mm, and 18 mm, respectively. A scarp and steep face developed at the rear of the surcharge zone, signifying a complete loss of the slope’s bearing capacity. Overall, the failure mechanism involved stress concentration that initiated and propagated cracks, the formation of a potential slip surface, and ultimately toe bulging, shearing, and overall sliding failure.In summary, the formation and evolution of slope failure under applied loading follows a “progressive migration” mechanism: loading → initial equilibrium disruption → rear-edge tensile cracking → upper soil sliding → front-edge compression and bulging → sliding surface propagation and interconnection → overall slope sliding failure."

②The regression equations (Eg 1 and Eq 2) are presented without sufficient context. The R-squared values or other goodness-of-fit metrics are essential to judge their validity. The units for factors A, B, and C in Eq 1 are missing.

Thanks for your comments. We have provided a detailed supplement to this section. In Section 2.1.2, we incorporated a range analysis to further elucidate the influence patterns of each factor on the similar materials. In Section 2.1.3, we added the fitted curve plots and the corresponding R2 values, and clarified the units used in the regression equations.

"**2.1.2 Influence Mechanisms of Various Factors on Similar Materials**

This study employed the range analysis method to examine how the four component factors A, B, C, and D influence the material’s physical-mechanical parameters and hydraulic properties.The range is calculated as the difference between the maximum and minimum average values across the levels of each factor.A larger range indicates that the corresponding factor exerts a stronger influence on the properties of the similar materials.

**2.1.2.1 Analysis of Factors Affecting the Density of Similar Materials**

Based on the orthogonal test results, a range analysis was performed to evaluate the density of the similar materials.The average values for each level of factors A-C were calculated, and the corresponding line chart was plotted, as shown in Fig 8.The results indicate that the relative influence of the factors on the density of the similar materials decreases in the order of B (range: 0.077, with subsequent values also representing ranges)> A (0.0575)> and C (0.0503).Factor B (barite powder/aggregate) is the primary factor controlling the density.A lower barite powder content combined with higher contents of river sand and heavy calcium powder leads to an increase in the density of the similar materials.This finding further confirms that incorporating river sand and heavy calcium powder into the aggregate can effectively regulate the unit weight of similar materials.

**Fig. 8 Analysis of density sensitivity factors**

**2.1.2.2 Analysis of Factors Affecting the Cohesion and Internal Friction Angle of Similar Materials**

Based on the test results for each group of similar materials, the average cohesion and internal friction angle corresponding to each factor were calculated under both natural and water‑saturated conditions, and the resulting line chart is presented in Fig 9.

**Fig. 9 Analysis of sensitivity factors affecting cohesion and internal friction angle**

Based on the calculations, the influence of each factor on cohesion is ranked as A (0.405) > C (0.350) > B (0.330).This result indicates that the sensitivity of cohesion is primarily governed by factor A (river sand/aggregate).As the river sand content increases, the interlocking and contact characteristics among the particles of the similar materials are altered, promoting the formation of a more stable internal structural framework that enhances cohesion.Although double fly ash powder and barite powder also exert an influence on cohesion, their effects are weaker compared with the changes induced by variations in aggregate particle gradation.These findings suggest that the cohesion of similar materials is predominantly controlled by the configuration of the aggregate skeleton, while fine‑grained materials mainly serve as fillers and provide localized cementation.

Similarly, the influence of each factor on the internal friction angle is ranked as B (2.14) > C (1.07) > A (0.74).Among these factors, factor B (barite powder/aggregate) exerts the most significant and dominant control over the internal friction angle.Variations in barite powder content lead to substantial changes in particle morphology, compactness, and interparticle friction within the similar materials, resulting in pronounced differences in their frictional behavior.Barite powder is characterized by its high density and relatively smooth, low‑angularity particles; therefore, when its content decreases, the rougher particle surfaces formed by river sand and double fly ash powder, together with increased aggregate contact strength, contribute to an increase in the internal friction angle.It is also notable that factor C (double fly ash powder/aggregate) has a measurable influence on the internal friction angle, primarily because double fly ash powder fills the voids within the skeleton and adjusts particle packing, thereby indirectly modifying the material’s overall shear‑resistance system.

In summary, cohesion is primarily governed by the structural characteristics of the aggregate particle framework, whereas the internal friction angle is mainly controlled by interparticle frictional resistance and material compactness.The differing sensitivities of these two strength parameters to the various factors reflect the distinct dominant mechanisms associated with internal structure and particle gradation during the shear behavior of similar materials.These results provide a reliable basis for optimizing mixture proportions and conducting subsequent model tests involving similar materials.

**2.1.3 Multivariate Regression Analysis of Parameters and Determination of Experimental Material Mix Proportions**

In this study, multivariate regression analysis was conducted to quantify the relationships between each physical and mechanical parameter and the compositional proportions of the three factors A, B, and C.The fitting results for each parameter are presented in Fig 10.The coefficients of determination between the regression‑derived fitted values and the corresponding experimental values range from $R_{a}^{2}$ = 0.8732 to 0.9326, indicating a strong goodness of fit.The regression equations are provided in Eq 5.

(5)

In the equation, a represents the percentage of river sand mass relative to the aggregate mass; b represents the percentage of barite powder mass relative to the aggregate mass; and c represents the percentage of double‑fly ash powder mass relative to the aggregate mass.

**Fig. 10 Comparison of the multivariate regression fitting results with the experimental results for each parameter"**

4. Discussion and Novelty:

The discussion section effectively synthesizes the findings but could be strengthened by a more direct comparison with existing literature. The introduction rightly identifies a gap regarding "stress disturbances caused by engineering activities" (line 77). The discussion should explicitly state how the discovered "progressive migration" mechanism (line 435) differs from failure mechanisms triggered by rainfall or earthquakes, as studied by the cited authors (Khan, Gan, Ma, etc.). This would better highlight the manuscript's novel contribution.

Thanks for your comments. We added Section 5 to conduct a comparative analysis. In Section 5.1, we review the failure mechanisms and characteristics of homogeneous slopes subjected to additional loading. In Section 5.2, we compare the failure mechanism proposed in this study with those of homogeneous slopes under additional loading, as well as with the failure mechanisms of colluvial slopes under rainfall and seismic conditions and of rock slopes. Due to the structural features of colluvial deposits and the discontinuous nature of joints, the failure mechanism of colluvial slopes under surcharge loading indeed exhibits distinctive characteristics.

"5.Comparative analysis

**5.1 Studies for comparison with the present work**

To validate the rationality of the deformation and failure mechanism proposed in this study and to highlight the distinct deformation and failure characteristics of accumulation slopes, Table 12 compiles previous research on slope deformation and failure under additional loading for comparative analysis.

**Table 12. Comparison of slope deformation and failure modes under external loading**

| **Source** | **Category** | **Load type** | **Loading position** | **Research method** |
| --- | --- | --- | --- | --- |
| [57] | Homogeneous slope | Uniform load, trapezoidally distributed load and concentrated load | Slope crest | Numerical analysis |
| [58] | Homogeneous slope | Uniform load, embedded load, concentrated load | Slope crest | Numerical analysis combined with experimental testing |

Ref. [57] examined the deformation and failure behavior of slopes subjected to additional loading using numerical analysis. The deformation-failure pattern under a uniform load is illustrated in Fig. 27a. The findings show that, once a uniform load is applied to the slope crest, the slope initially enters an elastic response stage, during which overall deformation remains small and no clear indicators of failure emerge. With continued increases in load magnitude, a shear strain concentration zone develops in the shallow region near the slope crest and gradually evolves into a localized shear band, marking the transition from the elastic stage to the elasto-plastic stage. During this stage, the shallow shear band extends in a strip-like form along the slope surface, representing a characteristic feature of the initial failure process.

As the external load continues to increase, the shallow shear band progressively extends downward and eventually connects to form a continuous weak shear surface, accompanied by a marked enlargement of the plastic zone within the slope. The failure zone follows a characteristic shallow‑to‑deep evolutionary pattern: it initiates with localized damage in the shallow portion of the slope surface, advances gradually into the deeper interior, and ultimately develops into a deep‑seated sliding surface. This progression is also reflected in the displacement-load curve, which exhibits a rapid increase in slope as the load approaches its ultimate value, indicating a significant reduction in slope stiffness and the onset of accelerated instability. Displacement contours from the numerical simulation further demonstrate an overall sliding deformation extending from the slope crest to the toe, with deep‑seated mass movement constituting the dominant failure mode. Failure induced by a uniform load is characterized by a large affected zone, parallel shear‑band development, and a progressively deepening slip surface, highlighting the broad and integral nature of slope failure under uniform loading.

**Fig 27 Failure modes of a homogeneous slope subjected to uniform loading: (a) Failure mode of the slope under a uniform load; (b) Failure mode of the slope under a locally applied uniform load.**

Ref. [58] examined the three‑dimensional slope stability and associated failure mechanisms when a locally applied uniform load was imposed at the slope crest. The deformation and failure pattern under the local uniform load is shown in Fig. 27b. The findings indicate that, unlike the widespread shallow failures induced by a fully uniform load, the slope failure triggered by a local load exhibits pronounced localization and directionality. During the initial loading stage, a distinct stress concentration and the onset of a plastic zone first occur beneath the loaded area, manifested in the rapid development of a localized shear band. The peak shear strain within this zone is substantially higher than that in the surrounding soil, causing the local structural integrity of the slope to be compromised first.

As the load continues to act and the plastic zone expands, the shear band progresses into the slope interior and downward in a wedge‑shaped or tongue‑shaped pattern, exhibiting pronounced three‑dimensional propagation characteristics. Because the loaded area is limited, the failure no longer develops uniformly along the slope surface but instead evolves into an asymmetric shear‑failure path aligned with the direction of stress concentration. The local load also tends to induce tensile cracking at the slope crest. These cracks widen as deformation increases and gradually propagate toward the slope surface, with their trajectory consistent with the contour of the internal shear band, further confirming the localized and directional nature of the failure.

When the localized shear zone expands to a critical scale, the plastic zone extends toward the slope toe and becomes fully connected, forming a composite shear‑sliding surface and marking the onset of overall slope instability. At this stage, the displacement curves show a rapid increase in the displacement difference between the upper and lower segments of the sliding surface, indicating that the sliding mass is transitioning into an accelerated downslope movement phase.

Compared with a uniformly distributed load, failure induced by a local load is more strongly governed by stress‑concentration points. Although the affected zone is relatively limited, the resulting damage is more intense, making rapid localized failure more likely and allowing it to propagate progressively to the entire slope. Overall, under a locally applied uniform load, slope failure exhibits features such as point‑to‑surface evolution, pronounced spatial directionality, and three‑dimensional wedge‑shaped expansion of the shear band, demonstrating the strong perturbation effect of local loading on slope stability.

**5.2 Comparative analysis results**

**5.2.1Comparison of Failure Mechanisms in Homogeneous Slopes under Additional Loading**

This study compares the slope failure patterns identified herein with those reported in previous studies under uniformly distributed loads and locally applied uniformly distributed loads. The results show that slopes composed of colluvial or accumulative deposits exhibit a characteristic “pushing‑type” failure mode when subjected to surcharge‑induced disturbances. These failure features arise not only from the inherent properties of the deposits—namely weak cementation and low shear strength—but also from the pronounced influence of load type, stress‑transfer pathways, and modes of spatial propagation.

At the initial stage of failure evolution, the slope was primarily undergoing stress redistribution, characterized by the emergence of shallow short cracks, the formation of tensile cracks at the rear edge of the surcharge, and localized settlement near the slope toe. The characteristics of this stage align with the early deformation patterns reported under uniformly distributed loading in Ref. [57], which similarly show that shallow stress perturbations develop first. However, unlike the planar and uniformly applied disturbance produced by a uniformly distributed load, the surcharge in this study functions as an approximately “locally concentrated load.” Consequently, the rear‑edge tensile cracks are more pronounced and display distinct structural‑control features, more closely matching the initiation behavior of localized shear zones induced by locally applied uniformly distributed loads as described in Ref. [58].

As the surcharge continues to act, the slope transitions into a stage of intensified deformation. The number and width of cracks increase markedly, and the crack network expands both inward and laterally within the slope. Penetrating microcracks develop within the interior and gradually evolve into a potential slip surface. This process is consistent with the “shallow-intermediate-deep progressive extension” failure pathway reported under uniformly distributed loading, reflecting the gradual formation of the slip surface. However, the colluvial slope simultaneously exhibits a pronounced coupled response characterized by frontal bulging and rear‑edge downward displacement. The relative movement of the sliding mass becomes more evident, representing a typical pushing‑type structural failure. In contrast, shear bands formed under uniformly distributed loads tend to be more parallel and widespread, whereas those generated by localized loading display stronger directionality and wedge‑shaped propagation. Therefore, the failure mode observed in this study falls between the global extension produced by uniformly distributed loads and the directional extension induced by localized loads, but it more closely resembles failure patterns triggered by localized loading.

At the final failure stage, all three types of slopes exhibit a fully developed slip surface and overall instability of the sliding mass. Under uniformly distributed loading, the slip surface penetrates deeply and continuously, producing large‑scale downslope displacement. Under localized loading, the shear surface typically shows three‑dimensional wedge‑shaped or tongue‑shaped propagation. The pushing‑type failure identified in this study is characterized by a distinct rear scarp, a bulging shear‑out zone at the slope front, and a through‑going slip surface that develops from top to bottom, ultimately forming an integrated sliding accumulation mass. Although this failure mode aligns more closely with the directional failure induced by localized loading, its progressive evolution is more complex, exhibiting a multi‑stage coupled sequence: initiation of rear‑edge tensile cracking → sliding of the upper‑middle block → frontal shear‑bulging extrusion → penetration of the slip surface → overall sliding.

Overall, the failure mechanism of colluvial slopes is jointly governed by the structural characteristics of the colluvial material, the looseness of joints, and the strong stress‑concentration effects induced by localized surcharge loading. In terms of failure evolution, the slopes exhibit both the “shallow‑to‑deep” shear‑extension pattern typical of uniformly distributed loading and the “point‑to‑surface” wedge‑shaped directional propagation associated with localized loading. However, the final evolution of colluvial landslides tends to develop into a typical pushing‑type failure, in which the failure process is more strongly influenced by the surcharge location, surcharge width, and the stratification of the colluvial deposits.

Therefore, the failure mechanism of colluvial slopes under surcharge loading cannot be simply categorized as either the uniformly distributed loading-induced mode or the localized loading-induced mode. Rather, it should be understood as a surcharge‑induced cumulative, pushing‑type, and structurally controlled failure mechanism that develops on the basis of the inherent structural weakness of the colluvial deposits.

**5.2.2 Comparison of Failure Mechanisms in Colluvial Slopes under Rainfall and Seismic Loading**

For the rainfall‑induced failure of colluvial deposits, the comparative reference used in this study is Ref. [16]. Khan et al. investigated the failure mechanism of unsaturated coal‑gangue colluvial slopes subjected to rainfall infiltration [16]. The failure process induced by rainfall infiltration in coal‑gangue slopes evolves through two distinct stages. The first stage is “seepage‑driven saturation and suction loss,” during which rainwater rapidly infiltrates through the highly permeable gangue material, causing matric suction to decrease sharply as the water content increases. Due to its highest permeability, the slope crest becomes saturated first, leading to nonuniform swelling and the formation of tensile cracks. The second stage is “strength degradation and initiation of sliding.” As seepage continues, pore‑water pressure shifts from negative to positive, effective stress progressively decreases, and the shear strength along the potential slip zone significantly deteriorates. Under the combined effects of self‑weight and seepage forces, a traction‑type sliding initiates from the crest and propagates downslope, ultimately reducing the factor of safety to below 0.9 and resulting in global slope failure.

For earthquake‑induced failures in colluvial deposits, Ref. [23] is used as the comparative source in this study. Ma et al. examined the failure mechanisms of colluvial slopes through shaking‑table experiments [23]. The seismic failure process of colluvial‑layer landslides can be summarized in two stages. The first stage is “crack initiation and propagation.” As seismic waves travel upward from the base of the slip zone, the combined effects of slip‑zone strength softening and topographic amplification lead to the formation of tensile cracks in the mid‑slope region. These cracks then propagate toward the slope toe, outlining the initial geometry of a throughgoing slip surface. The second stage is “slip‑surface formation and instability onset.” With continued shaking, the soil within the slip zone undergoes plastic yielding, the point safety factor decreases overall and exhibits a delayed peak, and a deep tensile crack forms at the rear of the sliding mass. The sliding mass subsequently undergoes a pushing‑type displacement from the mid‑slope toward the slope toe, ultimately resulting in global failure as the shear resistance along the slip zone drops sharply.

The failure mechanisms of colluvial slopes under different external disturbances exhibit both distinct differences and shared characteristics. Under rainfall conditions, landslide failure progresses through two stages: rainwater first infiltrates rapidly through highly permeable zones, causing early saturation at the slope crest and the development of tensile cracks; subsequently, rising pore‑water pressure leads to strength degradation along the slip zone, inducing traction‑type sliding from the crest toward the toe. Under seismic loading, failure initiates with tensile cracking in the mid‑slope region, after which the slip zone undergoes plastic yielding, and the sliding mass experiences a pushing‑type displacement from the mid‑slope toward the slope front. In contrast, slope failure induced by surcharge loading in this study exhibits a typical “pushing‑type” evolution. Surcharge‑driven stress redistribution leads sequentially to rear‑edge tensile cracking, crest sliding, and frontal bulging, during which the potential slip surface progressively develops and ultimately forms a tongue‑shaped sliding mass.

From a mechanistic perspective, rainfall‑induced landslides are primarily controlled by increases in pore‑water pressure and the degradation of shear strength, whereas earthquake‑induced landslides are driven by instantaneous shearing and plastic yielding. In contrast, surcharge‑induced landslides are triggered by stress accumulation and localized stress concentration, exhibiting progressive failure with pronounced structural‑control features. The failure process under surcharge loading involves both the “shallow‑to‑deep” shear propagation characteristic of uniformly distributed loads and the wedge‑shaped “point‑to‑surface” propagation induced by localized loads, while more prominently displaying a multistage interaction consisting of rear‑edge tensile cracking, translational sliding of the upper slope, and frontal bulging. Therefore, the failure mechanism of colluvial slopes subjected to surcharge loading is unique: it differs from the traction‑type sliding induced by rainfall and from the pushing‑type instability caused by earthquakes, instead manifesting as a cumulative-pushing-structurally controlled composite failure mode.

**5.2.3 Failure Mechanisms in Comparison with Rock Slopes**

In addition, colluvial slopes differ markedly from rock slopes, with their failure patterns more strongly governed by material weakness and structural looseness. Because rock masses possess higher structural integrity and more regular joint-fracture networks, the failure of rock slopes is typically controlled by geometrically well‑defined combinations of structural planes, resulting in structural instability modes such as block sliding, wedge failure, or toppling, with the failure surface being relatively predictable [59]. In contrast, colluvial slopes consist of rock fragments, soil materials, and loose accumulations, and their internal structure is highly heterogeneous. Their deformation generally develops through progressive plastic propagation, and their sliding surfaces tend to be more tortuous, less continuous, and more susceptible to local disturbances. Compared with the “structure‑controlled overall sliding” commonly observed in rock‑slope failures, colluvial slopes more readily initiate microcracks at sites of local stress concentration and progressively evolve along weak, poorly persistent zones under sustained surcharge loading, ultimately forming a pushing‑type overall slide.

Meanwhile, the differing responses of rock slopes under uniformly distributed loading and local loading are mainly reflected in the extent to which structural planes participate in deformation and in the geometric variations of shear bands. In contrast, because particle contacts within colluvial slopes are inherently loose, local surcharge loading more readily induces pronounced stress‑concentration effects, resulting in coupled deformation characterized by rear‑edge tension cracking, front‑edge bulging, and segmented displacement of the sliding mass. This leads to an overall evolutionary pattern of “zonal response-coupled failure” [59].

In summary, from an engineering perspective, colluvial slopes show pronounced failure sensitivity under surcharge loading. The progressive development of rear‑edge tension cracking, front‑edge bulging, and deep shear bands should be regarded as key monitoring indicators. Controlling surcharge intensity, limiting the spatial extent of surcharge loading, mitigating stress concentration at the surcharge-slope interface, and improving the shear strength of both shallow and deep layers of the colluvial deposits are essential measures for preventing surcharge‑induced landslides. In landslide‑prone regions, dynamic monitoring systems can be employed for continuous observation [60]."

**Minor Points and Editorial Corrections**

1.Abstract: The abstract is well-written but could be slightly more specific. For example, "greatest at the slope crest" could be "up to X mm at the slope crest. "

Thanks for your comments. We incorporated quantitative descriptions into the abstract.

"Large-scale artificial loading often triggers landslides in colluvial deposits, yet the mechanical response and failure mechanisms of such slopes under loading remain insufficiently understood. Using a typical colluvial slope in Tibet as a case study, this research integrates physical model tests and FLAC3D simulations to investigate loading-induced deformation and failure processes. Analogous materials composed of river sand, barite powder, calcium carbonate powder, and water were prepared, and multiple regression analysis was used to establish empirical relationships between mix ratios and the resulting cohesion and internal friction angle, yielding high similarity ($R_{a}^{2}$= 0.8732-0.9326). Under loading, the slope exhibits maximum vertical and horizontal displacements of 40 mm and 50 mm, respectively, with shear stress concentrated along the loading boundary and vertical stress penetrating deeper than horizontal stress. ......"

2.Keywords (line 38): The keywords are run together. They should be separated by semicolons for proper indexing: "External Loading; Deposit slope; Physical Model Experiment; Slope Deformation and Failure"

Thanks for your comments. We have completed the revisions.

"Keyword：Artificial Surcharge Loading; Colluvial Slope; Physical Model Experiment; Slope Deformation and Failure"

3.Author Contribution (lines 466-468): The formatting is inconsistent. lt should be standardized, e.g., Pei Zuan: Conceptualization, Data curation, Writing - original draft, Investigation, Supervision.

Thanks for your comments. We have standardized the format.

"Pei Zuan: Conceptualization, Data curation, Writing-original draft, Investigation, Supervision.

Jiali Feng: Conceptualization, Data collection, Writing-final draft, Visualization, Investigation.

Fenglin Ren: Conceptualization, Data collection, Writing-final draft, Software."

4.Line 110-115: The stratigraphic nomenclature (Q4[m], Q4pφ, Q4[de]) is confusing. The symbol "φ" is unusual, and the use of brackets and superscripts is inconsistent. Please use a consistent format throughout.

Thanks for your comments. We have made the corresponding revisions in the manuscript.

"The stratigraphic sequence of the slope, from youngest to oldest, consists of: Quaternary Holocene artificial fill ($Q_{4}^{ml}$), Holocene cultivated soil ($Q_{4}^{pd}$), Holocene landslide deposits($Q_{4}^{del}$), Holocene colluvial-alluvial deposits ($Q_{4}^{al+pl}$), and Upper Pleistocene glaciofluvial deposits ($Q_{3}^{fgl}$). Based on the results of the geotechnical survey, the slope is primarily composed of three strata: $Q_{4}^{del}$, $Q_{4}^{al+pl}$, and $Q_{3}^{fgl}$. The main materials include cobble-bearing soil, cultivated soil, and plain fill. The engineering geological cross-section is shown in Fig 2."

5.Line 199-204: The description of the model box and loading is good, but the size of the sandbags (1-meter-long) seems to be a prototype-scale description leaking into the model-scale description. Clarify.

Thanks for your comments. A 1‑meter sandbag was used as the applied load in the model tests. In the model test design, the model box measured 1.6 m × 1 m × 1.6 m, and the 1‑meter sandbag precisely covered the width of the box.

6.Language and Grammar: The manuscript is generally well-written but would benefit from a thorough proofread by a native English speaker to correct minor grammatical awkwardness(e.g. "Sensor 5 exceeding readings..." in line 300).

Thanks for your comments. We have made improvements in response to the issues you raised.

Reviewer 2:

Comments to the Author

The introduction provides a strong global context on medical geology and the role of a sustainable national framework. This work shows the authors' understanding of international best practices. However, the section sometimes lacks organizational flow, with abrupt transitions between the data, technologies, and theoretical models.

The Objectives and Methodology section presents a relevant goal. However, the methodology lacks critical detail-there is no clear explanation of search strategies. databases used, selection processes, or thematic coding. A structured PRlSMA-style flowchart or summary table should be added for clarity. inclusion and exclusion criteria are vaguely addressed, and no justification is given for the types of studies.

The Results and Findings section is well organized. However, it needs to integrate quantitative data (casualties, damages) with qualitative assessments and include useful visuals like photos and maps. The conceptual framework and research questions are poorly written. Furthermore, the research needs to be attractive.

The data collection and data analysis in this paper are relatively concise, and the author is advised to enrich this part of the content.

1. General Comment:

The authors may consider making their paper more concise and focusing more in the Landslide and slope Stability component they wish to introduce or highlight. The rationale behind the introduction of this framework needs to be clearly established. In addition, providing a conceptual framework on landslides would enhance appreciation and understanding of the framework.

Thanks for your comments. We streamlined the corresponding sections; however, to enhance clarity and provide useful references for future researchers, we also incorporated essential details, including parameter selection, mix proportions of the analogous materials, and the fitted equations.

2. Introduction:

The authors did not explicitly present the rationale of the study, Some of the information provided is scattered and needs to be stated more logically and cohesively.

It would also strengthen the paper if the objectives of the study were clearly mentioned.

Further explanation of landslides and slope Stability is essential, particularly in demonstrating how it is more encompassing than the "landslide" theme.

Thanks for your comments. We revised the introduction section and offered a more detailed description of the research objectives.

"**Introduction**

......As an important external load commonly encountered in mountainous transportation engineering, mining operations, and land‑use development [24], surcharge loading is characterized by sudden application, concentrated distribution, and high intensity [25-27]. Such loading can substantially alter the stress and seepage fields of slopes [28], accelerate the accumulation of local damage and the formation of slip surfaces, and consequently cause the failure mode and instability evolution to differ significantly from those under natural conditions [29]. However, existing studies provide insufficient attention to this issue, and systematic investigations into the deformation response, failure‑process evolution, and controlling mechanisms of accumulation‑body slopes under surcharge loading remain limited. This research gap is particularly pronounced in plateau mountainous regions, where large topographic relief and complex rock-soil structures further complicate slope behavior.

To address these gaps, this study investigates a typical deposit slope in Tibet, integrating laboratory physical model experiments with numerical simulations to systematically examine slope deformation and failure characteristics under external loading, while revealing the underlying instability mechanisms. The results provide a theoretical foundation for the siting of major infrastructure and slope-support design, as well as technical guidance for enhancing early detection and risk management of deposit slope failures. "

[24] Zhang L, Wang Y, Chen H, Li X, Zhou J, Liu M, et al. Study on slope failure evolution under surcharge loading and toe cutting with BOTDA technology. Opt Fiber Technol. 2021; 66:102644. <https://doi.org/10.1016/j.yofte.2021.102644>.

[25] [Manna B, Biswas S, Roy P, Saha D, Ghosh A, Mukherjee S, et al. Effect of surcharge load on stability of slopes - testing and analysis. Electron J Geotech Eng. 2014; 19:3397-410.](https://www.researchgate.net/publication/289186804_Effect_of_surcharge_load_on_stability_of_slopes_-_testing_and_analysis)

[26] Xu C, Li Y, Zhang H, Chen F, Wang J, Zhou L, et al. Numerical analysis of surcharge effect on stability and interaction mechanism of slope-pile-footing system. J Earth Sci. 2024;35(3):955-69. <https://doi.org/10.1007/s12583-023-1866-7>.

[27] Li N, Cheng YM. Laboratory and 3-D distinct element analysis of the failure mechanism of a slope under external surcharge. Nat Hazards Earth Syst Sci. 2015;15(1):35-43. <https://doi.org/10.5194/nhess-15-35-2015>.

[28] Cheng YM, Li N, Zhang L, Wang H, Chen J, Liu Z, et al. Three-dimensional slope stability problem with a surcharge load. Nat Hazards Earth Syst Sci. 2015;15(10):2227-40. <https://doi.org/10.5194/nhess-15-2227-2015>.

[29] Roy S, Singh A, Kumar P, Sharma R, Patel M, Gupta S, et al. Analysis of unsaturated slope stability under seismic and surcharge loading by upper bound rigid block method. arXiv. 2024; arXiv:2407.16633. <https://doi.org/10.48550/arXiv.2407.16633>.

3. Materials and Methods:

This section appears to be an extended well and actual materials or methods used in the

study were presented.

Thanks for your comments. We made more detailed revisions in this section. In Section 2, we added a flowchart and further supplemented the configuration details and specific mix proportions of the analogous materials.

"2. Research Methodology

The primary research methods used in this study are laboratory model testing and numerical simulation. First, an indoor physical model test was conducted by preparing similar materials based on data obtained from field investigations and geological surveys. In view of the inherent limitations of physical model testing, numerical simulations were subsequently performed according to similarity relationships, using the prototype dimensions along with the corresponding load levels and loading durations. Integrating these two approaches enables both comparative verification and a deeper interpretation of the deformation and failure mechanisms of accumulation‑body slopes under surcharge loading. The overall workflow is illustrated in Fig. 3

**Fig. 3 Overall workflow of the research methodology**

**2.1** **Physical Model Test**

Due to the high cost, long duration, and limited controllability of field experiments [30], as well as the difficulty of reproducing specific environmental conditions, this study employs laboratory-scale physical model experiments, which offer high controllability, reproducibility, and relatively low cost [31]. The experimental procedure is illustrated in Fig 4.

**Fig. 4 Experimental procedure flowchart**

**2.1.1 Similarity Material Proportioning**

The development of analogous materials is a prerequisite for conducting physical model experiments and is a critical factor in determining whether the model can accurately replicate the physical properties of the prototype [32]. Based on similarity theory, this study establishes quantitative relationships between the model and the prototype to reveal the underlying mechanisms of complex phenomena. To ensure that the model’s deformation behavior corresponds to that of the actual engineering system, the model must satisfy the required similarity criteria. All relevant parameters can be represented by the following relationship, as shown in Eq 1.

(1)

In Eq 1, *l* denotes the model size, *γ* is the unit weight of the model, *g* represents gravitational acceleration, *c* is cohesion, *φ* is the internal friction angle, *E* is the elastic modulus of the model, δ is displacement, *σ* is stress, *ε* is strain, *μ* is Poisson’s ratio, *t* is time, and *F* is force. Because *φ, ε*, and *μ* are dimensionless quantities, their similarity ratios are equal to 1, that is, *Cφ* = *Cμ* = *Cε* = 1. When *Cγ* is set to 1, the derivation of the remaining physical quantities must satisfy the similarity criteria specified in Eqs 2-4.

$C_{l}=l_{p}/l_{m}$ (2)

In the equation, *C_l_* is the geometric similarity constant with the dimension of length, where the subscript *p* denotes the prototype and the subscript m denotes the model.

$\left\{\begin{matrix} C_{\sigma}=C_{l}C_{\gamma} \\ C_{\sigma}=C_{E}C_{\varepsilon} \\ C_{\delta}=C_{l} \\ C_{F}=C_{\sigma}C_{l}^{2} \end{matrix}\right.$ (3)

In the equation, *C_σ_* denotes the stress similarity constant, *C_γ_* represents the unit‑weight similarity constant, *C_E_* is the elastic modulus similarity constant, *C_ε_* is the strain similarity constant, *C_δ_* is the displacement similarity constant, and *C_F_* is the force similarity constant.

$\left\{\begin{matrix} C_{\sigma}=C_{R}^{\tau} \\ C_{c}=C_{R}^{\tau} \\ C_{R}^{c}=C_{R}^{\tau} \end{matrix}\right.$ (4)

In the equation, $C_{R}^{\tau}$ denotes the tensile strength similarity constant, $C_{c}$ represents the cohesion similarity constant, and $C_{R}^{c}$ is the compressive strength similarity constant.

The principal geometric dimensions of the debris‑slope cross‑section are shown in Fig 5. To ensure experimental operability, the geometric similarity constant was set to 200 in the model tests, and the similarity ratios for the other physical quantities are provided in Table 1.

**Fig. 5 Geometric dimensions of the debris‑slope cross‑section**

**Table 1. Similarity parameters between model and prototype**

| **Physical Quantity** | **Dimension** | **Similar Quantity** | **Similarity Ratio** |
| --- | --- | --- | --- |
| Geometric length (*L)* | L | $C_{L}$ | 200 |
| Internal friction ($\varphi$) | 1 | $C_{\varphi}$ | 1 |
| Cohesion (*c*) | FL^-1^T^-2^ | $C_{c}$ | 200 |
| Water content (*ω*) | 1 | $C_{\omega}$ | 1 |
| Unit weight (*γ*) | FL^-3^ | *C_γ_* | 1 |
| Strain (*ε*) | 1 | *C_ε_* | 1 |
| Elastic modulus (*E*) | FL^-2^ | *C_E_* | 200 |
| Poisson’s ratio (*μ*) | 1 | C*_μ_* | 1 |
| Displacement (*δ*) | L | C*_δ_* | 200 |
| Stress (*σ*) | FL^-2^ | C*_σ_* | 200 |
| Force (*F*) | F | *C_F_* | 200^3^ |
| Flexural rigidity (*EI*) | FL^2^ | C*_EI_* | 200^5^ |
| Time (*t*) | T | *C_t_* | 200^1/2^ |

The soil’s mechanical parameters were determined through laboratory testing, as summarized in Table 2. The testing procedure is shown in Fig 6. The natural density of the soil was measured using the cutting‑ring method, while the maximum dry density and optimum moisture content were obtained from compaction tests. Cohesion, internal friction angle, deformation modulus, and Poisson’s ratio were derived from large‑scale triaxial tests. Detailed testing procedures are provided in Appendix A.

**Fig. 6 Procedure for obtaining soil mechanical parameters**

**Table 2. Main mechanical parameters of the slope soil**

| **Stratum** | **Soil Type** | **Natural Density (g/cm^3^)** | **Deformation Modulus (MPa)** | **Poisson’s Ratioμ** | **Cohesion**  **(kPa)** | **Internal Friction Angle**  **(°)** |
| --- | --- | --- | --- | --- | --- | --- |
| Q_4_^del^ | Accumulated gravelly soil | 2.02 | 24.5 | 0.3 | 8.7 | 31.0 |
| Q_3_^fgl^ | Fluvio-glacial gravelly sand | 2.07 | 24.5 | 0.3 | 9.3 | 32.1 |
| Q_4_^al+pl^ | Ice-water accumulated gravelly soil | 2.09 | 24 | 0.3 | 8.8 | 29.8 |

Based on the properties of in-situ soils and relevant literature [33-34], and considering the particle size effects in model experiments, the analogous materials were required to be silty and granular, with low cohesion and moderate-to-low strength. We reviewed the mix proportions of soil‑simulating materials reported in comparable studies, as summarized in Table 3.

**Table 3. Mix Proportions of Soil‑Simulating Materials in Comparable Studies**

| Serial Number | Analog Material System | Simulation Type | Source |
| --- | --- | --- | --- |
| 1 | Gypsum + Barite Powder + Bentonite + Sand | Accumulation with Weak Interlayers | [35] |
| 2 | Gypsum + River Sand + Barite + Cementing Agent | Layered Accumulation、Fractured Rock Mass、Stratified Slope | [36] |
| 3 | Barite Powder + Gypsum + Quartz Sand + Water | Stratified Slope、Sedimentary Sand Layer、Non‑Cohesive Depositional Soil | [37] |
| 4 | Gypsum + Fly Ash/Iron Powder + Barite + Loose Sand | Large‑Deformation Accumulation、Weathered Material Accumulation | [38] |
| 5 | River Sand + Barite + Gypsum + Cementing Agent | Generic Accumulation Simulation | [39] |
| 6 | Standard Sand + Barite Powder + Bentonite + Accumulation Soil | Man‑Made Accumulation Slope | [40] |
| 7 | Barite Powder + Quartz Sand + Gypsum + Glycerol + Water | Conventional Rock Slope | [41] |

As shown in Table 3, the mix materials that recur across various studies are primarily gypsum, barite powder, and quartz sand.Existing research indicates that barite powder is mainly used to adjust material density, quartz sand provides a granular framework or modifies the friction angle, and gypsum, as a commonly used cementing agent, enhances cohesion and overall strength.However, since the similar material in this study must exhibit powder‑like particle characteristics, gypsum is not suitable as a cementing component.A review of the relevant literature [42-45] shows that double fly ash powder is inexpensive and exhibits non‑hardening and non‑hydrating properties, making it well suited as an inert filler in weakly cemented or powder‑soil similar materials.Therefore, considering material accuracy, controllability, and economic feasibility, the final mix system selected in this study consists of barite powder, river sand, double fly ash powder, and water.

The experimental materials consisted of river sand, barite powder, double fly ash powder, and water, with their properties listed in Table 4. River sand served as the skeleton material, barite powder was used to adjust the model density, and double fly ash powder acted as fine filler to occupy voids and reduce cohesion.

**Table 4. Material parameters of the similarity model components**

| **Raw material** | **Appearance** | **Main component** | **Density/(g·m^-3^)** |
| --- | --- | --- | --- |
| River sand | Light yellow grains | Quartz>90% | 2.65 |
| Baryte powder | White powder | Barium sulfate>95% | 4.3 |
| Double fly powder(Ultrafine calcium carbonate powder) | White powder | Calcium carbonate>90% | 2.8 |

The data in this table were supplied by the vendor.

The proportioning experiments for the analogous materials were conducted using the orthogonal design method [46-49]. This method allows for the selection of uniformly distributed and representative schemes from all possible combinations of experiments while considering multiple influencing factors, thereby effectively reducing the number of experimental groups and efficiently analyzing the effects of each factor on the results [50]. In the proportioning experiments, three factors were considered: the mass ratio of river sand to aggregate (factor A), barite powder to aggregate (factor B), and doube fly ash powder to aggregate (factor C), each with five levels, resulting in a total of 15 experimental groups. To facilitate comparative analysis of the effects of different factors and to eliminate the influence of irrelevant conditions on the results, the moisture content of all analogous materials was fixed at 10% of the aggregate mass. The orthogonal design levels of the analogous materials are listed in Table 5.

**Table 5. Orthogonal design levels for similarity material composition**

| **Level** | **Factor A/%** | **Factor B/%** | **Factor C/%** |
| --- | --- | --- | --- |
| 1 | 50 | 30 | 20 |
| 2 | 55 | 25 | 20 |
| 3 | 60 | 25 | 15 |
| 4 | 65 | 20 | 15 |
| 5 | 70 | 20 | 10 |

Based on the orthogonal experimental design, the raw materials were prepared, samples were fabricated, and relevant parameters were tested. The experimental procedure is illustrated in Fig 7.

**Fig. 7 Experimental procedure for preparing and testing similarity materials**

The proportions and physical properties of the 15 sets of prepared analogous materials are presented in Table 6. Studies have shown that the shear strength parameters of geotechnical materials—cohesion *c* and internal friction angle $\text{φ}$ are key factors governing the slope safety factor. These parameters directly influence both the occurrence of slope failure and the potential failure mode, and thus should be treated as primary variables during material proportioning [51-54]. The results indicate that, within the selected proportioning range, the density ranged from 1.953 to 2.098 g/cm³, cohesion from 8.66 to 9.19 kPa, and the internal friction angle from 29.7° to 32.4°.

**Table 6. Results of similarity material mix tests. The numbers in the mix schemes represent the orthogonal design levels. Factors A, B, and C correspond to the mass ratios at each level.**

| **Test Number** | **Mixing scheme** | **Density (g/cm³)** | **Cohesion (kPa)** | **Internal friction angle (°)** |
| --- | --- | --- | --- | --- |
| S1 | 1：1：1 | 2.034 | 9.02 | 30.8 |
| S2 | 1：2：2 | 1.978 | 8.63 | 31.6 |
| S3 | 1：3：3 | 2.057 | 9.47 | 30.1 |
| S4 | 1：4：4 | 2.006 | 8.66 | 30.3 |
| S5 | 1：5：5 | 2.081 | 8.89 | 29.5 |
| S6 | 2：1：2 | 2.058 | 9.09 | 32.0 |
| S7 | 2：2：3 | 2.098 | 9.41 | 30.7 |
| S8 | 2：3：4 | 1.967 | 8.84 | 31.0 |
| S9 | 2：4：5 | 2.002 | 9.22 | 30.4 |
| S10 | 3：1：3 | 1.953 | 8.72 | 31.8 |
| S11 | 3：2：4 | 1.972 | 8.93 | 29.9 |
| S12 | 3：3：2 | 2.033 | 9.35 | 30.2 |
| S13 | 4：1：4 | 2.016 | 8.97 | 32.4 |
| S14 | 4：2：5 | 2.071 | 8.60 | 29.7 |
| S15 | 5：1：5 | 2.008 | 9.19 | 31.2 |

**2.1.2 Influence Mechanisms of Various Factors on Similar Materials**

This study employed the range analysis method to examine how the four component factors A, B, C, and D influence the material’s physical-mechanical parameters and hydraulic properties.The range is calculated as the difference between the maximum and minimum average values across the levels of each factor.A larger range indicates that the corresponding factor exerts a stronger influence on the properties of the similar materials.

**2.1.2.1 Analysis of Factors Affecting the Density of Similar Materials**

Based on the orthogonal test results, a range analysis was performed to evaluate the density of the similar materials.The average values for each level of factors A-C were calculated, and the corresponding line chart was plotted, as shown in Fig 8.The results indicate that the relative influence of the factors on the density of the similar materials decreases in the order of B (range: 0.077, with subsequent values also representing ranges)> A (0.0575)> and C (0.0503).Factor B (barite powder/aggregate) is the primary factor controlling the density.A lower barite powder content combined with higher contents of river sand and heavy calcium powder leads to an increase in the density of the similar materials.This finding further confirms that incorporating river sand and heavy calcium powder into the aggregate can effectively regulate the unit weight of similar materials.

**Fig. 8 Analysis of density sensitivity factors**

**2.1.2.2 Analysis of Factors Affecting the Cohesion and Internal Friction Angle of Similar Materials**

Based on the test results for each group of similar materials, the average cohesion and internal friction angle corresponding to each factor were calculated under both natural and water‑saturated conditions, and the resulting line chart is presented in Fig 9.

**Fig. 9 Analysis of sensitivity factors affecting cohesion and internal friction angle**

Based on the calculations, the influence of each factor on cohesion is ranked as A (0.405) > C (0.350) > B (0.330).This result indicates that the sensitivity of cohesion is primarily governed by factor A (river sand/aggregate).As the river sand content increases, the interlocking and contact characteristics among the particles of the similar materials are altered, promoting the formation of a more stable internal structural framework that enhances cohesion.Although double fly ash powder and barite powder also exert an influence on cohesion, their effects are weaker compared with the changes induced by variations in aggregate particle gradation.These findings suggest that the cohesion of similar materials is predominantly controlled by the configuration of the aggregate skeleton, while fine‑grained materials mainly serve as fillers and provide localized cementation.

Similarly, the influence of each factor on the internal friction angle is ranked as B (2.14) > C (1.07) > A (0.74).Among these factors, factor B (barite powder/aggregate) exerts the most significant and dominant control over the internal friction angle.Variations in barite powder content lead to substantial changes in particle morphology, compactness, and interparticle friction within the similar materials, resulting in pronounced differences in their frictional behavior.Barite powder is characterized by its high density and relatively smooth, low‑angularity particles; therefore, when its content decreases, the rougher particle surfaces formed by river sand and double fly ash powder, together with increased aggregate contact strength, contribute to an increase in the internal friction angle.It is also notable that factor C (double fly ash powder/aggregate) has a measurable influence on the internal friction angle, primarily because double fly ash powder fills the voids within the skeleton and adjusts particle packing, thereby indirectly modifying the material’s overall shear‑resistance system.

In summary, cohesion is primarily governed by the structural characteristics of the aggregate particle framework, whereas the internal friction angle is mainly controlled by interparticle frictional resistance and material compactness.The differing sensitivities of these two strength parameters to the various factors reflect the distinct dominant mechanisms associated with internal structure and particle gradation during the shear behavior of similar materials.These results provide a reliable basis for optimizing mixture proportions and conducting subsequent model tests involving similar materials.

**2.1.3 Multivariate Regression Analysis of Parameters and Determination of Experimental Material Mix Proportions**

In this study, multivariate regression analysis was conducted to quantify the relationships between each physical and mechanical parameter and the compositional proportions of the three factors A, B, and C.The fitting results for each parameter are presented in Fig 10.The coefficients of determination between the regression‑derived fitted values and the corresponding experimental values range from $R_{a}^{2}$ = 0.8732 to 0.9326, indicating a strong goodness of fit.The regression equations are provided in Eq 5.

(5)

In the equation, *a* represents the percentage of river sand mass relative to the aggregate mass; *b* represents the percentage of barite powder mass relative to the aggregate mass; and *c* represents the percentage of double‑fly ash powder mass relative to the aggregate mass.

**Fig. 10 Comparison of the multivariate regression fitting results with the experimental results for each parameter"**

4. Results:

For the statement, "The natural hazards from landslides pose..", it is suggested to simply use "hazards" since they are no longer natural in origin, in this context.

Under "Remediation Frameworks for landslide prone zones" no framework was presented. The authors may consider discussing other relevant frameworks and how landslides and slope Stability could address the gaps in those frameworks. It is also recommended that greater emphasis be placed on sections directly relevant to the proposed framework, while subsections that are less relevant need not be discussed in detail.

Thanks for your comments. I have revised and further refined the corresponding sections.

5. Discussion:

The discussion requires further improvement to comprehensively or concisely address the

results presented more extensively.

Thanks for your comments. We have enhanced the discussion section by incorporating a flowchart illustrating the failure mechanism and by comparing it with the mechanisms of rainfall‑induced landslides, earthquake‑induced landslides, and homogeneous slopes subjected to additional loading.

" 4. Discussion of Results and Summary of Deformation and Failure Mechanisms

**4.1 Discussion of Results**

Both experimental and numerical simulation results indicate that slope deformation under applied loading gradually propagates from the top toward the middle section. As the applied load increases, the high shear strain increment zones continuously migrate toward the slope middle, revealing a pronounced positive correlation between load magnitude and the spatial extent of shear deformation. The underlying mechanism is that the shear stress induced by the loading continuously intensifies, causing soil initially in an elastic or slightly damaged state to progressively enter the shear failure stage, thereby driving the evolution of the failure zone toward the slope middle and ultimately facilitating progressive instability.

Experimental results show that soil deformation is most pronounced near the loading area, a feature further confirmed by numerical simulations. Under the same load, the extent of shear failure decreases with increasing distance from the slope crest line, indicating that stress concentration is particularly pronounced near the crest, where soil more readily enters the shear failure stage and the failure zone forms first. Meanwhile, the slope toe experiences higher shear stress under the combined action of loading and self-weight. When this stress exceeds the soil’s shear strength, failure occurs and gradually propagates toward the slope middle. Consequently, the shear failure zones typically migrate simultaneously from beneath the loading area and from both ends of the slope toe toward the slope middle, eventually connecting over time to form a continuous failure band. This indicates that the slope crest line and the slope toe are the most sensitive regions for slope stability in the embankment.

In addition, the middle section of the slope with favorable free-face conditions exhibits more pronounced horizontal displacement. This is primarily attributed to insufficient lateral restraint under loading and the steeper terrain, which induces stress concentration and facilitates soil movement toward the free face. Simultaneously, the horizontal displacement directions of soil inside and outside the shear strain increment zone are opposite. This is due to significant differences in stress states: soil within the zone undergoes stronger compression and shear, resulting in horizontal displacements opposite to those of the soil outside the zone under combined gravitational effects.

**4.2 Embankment Slope Deformation and Failure under Surcharge Loading**

Based on physical model experiments and numerical analysis, this study summarizes the failure mechanism of deposit slopes under applied loading, which can be divided into three main stages, as shown in Fig 26.

**Fig. 26 Three stages of deformation and failure in an accumulation slope**

During the initial loading stage, the slope undergoes stress redistribution, with internal stresses in the soil not yet reaching shear strength. This stage is characterized by localized minor settlements and shallow cracks on the slope surface, with some soil blocks slipping. Local tensile cracks appear at the crest and front edge of the loading area, while soil at the rear edge experiences lateral compression, resulting in horizontal compression and shear deformation.

As time progressed and the surcharge load continued to increase, the maximum earth pressure at Monitoring Point No. 4 near the slope crest reached 1.88 kPa, while that at Monitoring Point No. 5 near the slope toe reached 1.85 kPa, exceeding the soil’s yield strength and triggering slope deformation and failure. The failure was mainly characterized by progressive crack propagation and penetration, accompanied by the formation of an internal slip surface. Uplift occurred at the slope toe within the surcharge zone, and Displacement Gauge No. 3 recorded a maximum horizontal displacement of 12 mm, indicating substantial lateral thrust that induced shear failure and produced a tongue‑shaped accumulation body. According to the displacement data, the maximum horizontal displacements at Points A3 (upper part of the slip belt), A2 (middle part), and A1 (lower part) were 7 mm, 50 mm, and 18 mm, respectively. A scarp and steep face developed at the rear of the surcharge zone, signifying a complete loss of the slope’s bearing capacity. Overall, the failure mechanism involved stress concentration that initiated and propagated cracks, the formation of a potential slip surface, and ultimately toe bulging, shearing, and overall sliding failure.In summary, the formation and evolution of slope failure under applied loading follows a “progressive migration” mechanism: loading → initial equilibrium disruption → rear-edge tensile cracking → upper soil sliding → front-edge compression and bulging → sliding surface propagation and interconnection → overall slope sliding failure.

**5. Comparative analysis**

**5.1 Studies for comparison with the present work**

To validate the rationality of the deformation and failure mechanism proposed in this study and to highlight the distinct deformation and failure characteristics of accumulation slopes, Table 12 compiles previous research on slope deformation and failure under additional loading for comparative analysis.

**Table 12. Comparison of slope deformation and failure modes under external loading**

| **Source** | **Category** | **Load type** | **Loading position** | **Research method** |
| --- | --- | --- | --- | --- |
| [57] | Homogeneous slope | Uniform load, trapezoidally distributed load and concentrated load | Slope crest | Numerical analysis |
| [58] | Homogeneous slope | Uniform load, embedded load, concentrated load | Slope crest | Numerical analysis combined with experimental testing |

Ref. [57] examined the deformation and failure behavior of slopes subjected to additional loading using numerical analysis. The deformation-failure pattern under a uniform load is illustrated in Fig. 27a. The findings show that, once a uniform load is applied to the slope crest, the slope initially enters an elastic response stage, during which overall deformation remains small and no clear indicators of failure emerge. With continued increases in load magnitude, a shear strain concentration zone develops in the shallow region near the slope crest and gradually evolves into a localized shear band, marking the transition from the elastic stage to the elasto-plastic stage. During this stage, the shallow shear band extends in a strip-like form along the slope surface, representing a characteristic feature of the initial failure process.

As the external load continues to increase, the shallow shear band progressively extends downward and eventually connects to form a continuous weak shear surface, accompanied by a marked enlargement of the plastic zone within the slope. The failure zone follows a characteristic shallow‑to‑deep evolutionary pattern: it initiates with localized damage in the shallow portion of the slope surface, advances gradually into the deeper interior, and ultimately develops into a deep‑seated sliding surface. This progression is also reflected in the displacement-load curve, which exhibits a rapid increase in slope as the load approaches its ultimate value, indicating a significant reduction in slope stiffness and the onset of accelerated instability. Displacement contours from the numerical simulation further demonstrate an overall sliding deformation extending from the slope crest to the toe, with deep‑seated mass movement constituting the dominant failure mode. Failure induced by a uniform load is characterized by a large affected zone, parallel shear‑band development, and a progressively deepening slip surface, highlighting the broad and integral nature of slope failure under uniform loading.

**Fig 27 Failure modes of a homogeneous slope subjected to uniform loading: (a) Failure mode of the slope under a uniform load; (b) Failure mode of the slope under a locally applied uniform load.**

Ref. [58] examined the three‑dimensional slope stability and associated failure mechanisms when a locally applied uniform load was imposed at the slope crest. The deformation and failure pattern under the local uniform load is shown in Fig. 27b. The findings indicate that, unlike the widespread shallow failures induced by a fully uniform load, the slope failure triggered by a local load exhibits pronounced localization and directionality. During the initial loading stage, a distinct stress concentration and the onset of a plastic zone first occur beneath the loaded area, manifested in the rapid development of a localized shear band. The peak shear strain within this zone is substantially higher than that in the surrounding soil, causing the local structural integrity of the slope to be compromised first.

As the load continues to act and the plastic zone expands, the shear band progresses into the slope interior and downward in a wedge‑shaped or tongue‑shaped pattern, exhibiting pronounced three‑dimensional propagation characteristics. Because the loaded area is limited, the failure no longer develops uniformly along the slope surface but instead evolves into an asymmetric shear‑failure path aligned with the direction of stress concentration. The local load also tends to induce tensile cracking at the slope crest. These cracks widen as deformation increases and gradually propagate toward the slope surface, with their trajectory consistent with the contour of the internal shear band, further confirming the localized and directional nature of the failure.

When the localized shear zone expands to a critical scale, the plastic zone extends toward the slope toe and becomes fully connected, forming a composite shear‑sliding surface and marking the onset of overall slope instability. At this stage, the displacement curves show a rapid increase in the displacement difference between the upper and lower segments of the sliding surface, indicating that the sliding mass is transitioning into an accelerated downslope movement phase.

Compared with a uniformly distributed load, failure induced by a local load is more strongly governed by stress‑concentration points. Although the affected zone is relatively limited, the resulting damage is more intense, making rapid localized failure more likely and allowing it to propagate progressively to the entire slope. Overall, under a locally applied uniform load, slope failure exhibits features such as point‑to‑surface evolution, pronounced spatial directionality, and three‑dimensional wedge‑shaped expansion of the shear band, demonstrating the strong perturbation effect of local loading on slope stability.

**5.2 Comparative analysis results**

**5.2.1 Comparison of Failure Mechanisms in Homogeneous Slopes under Additional Loading**

This study compares the slope failure patterns identified herein with those reported in previous studies under uniformly distributed loads and locally applied uniformly distributed loads. The results show that slopes composed of colluvial or accumulative deposits exhibit a characteristic “pushing‑type” failure mode when subjected to surcharge‑induced disturbances. These failure features arise not only from the inherent properties of the deposits—namely weak cementation and low shear strength—but also from the pronounced influence of load type, stress‑transfer pathways, and modes of spatial propagation.

At the initial stage of failure evolution, the slope was primarily undergoing stress redistribution, characterized by the emergence of shallow short cracks, the formation of tensile cracks at the rear edge of the surcharge, and localized settlement near the slope toe. The characteristics of this stage align with the early deformation patterns reported under uniformly distributed loading in Ref. [57], which similarly show that shallow stress perturbations develop first. However, unlike the planar and uniformly applied disturbance produced by a uniformly distributed load, the surcharge in this study functions as an approximately “locally concentrated load.” Consequently, the rear‑edge tensile cracks are more pronounced and display distinct structural‑control features, more closely matching the initiation behavior of localized shear zones induced by locally applied uniformly distributed loads as described in Ref. [58].

As the surcharge continues to act, the slope transitions into a stage of intensified deformation. The number and width of cracks increase markedly, and the crack network expands both inward and laterally within the slope. Penetrating microcracks develop within the interior and gradually evolve into a potential slip surface. This process is consistent with the “shallow-intermediate-deep progressive extension” failure pathway reported under uniformly distributed loading, reflecting the gradual formation of the slip surface. However, the colluvial slope simultaneously exhibits a pronounced coupled response characterized by frontal bulging and rear‑edge downward displacement. The relative movement of the sliding mass becomes more evident, representing a typical pushing‑type structural failure. In contrast, shear bands formed under uniformly distributed loads tend to be more parallel and widespread, whereas those generated by localized loading display stronger directionality and wedge‑shaped propagation. Therefore, the failure mode observed in this study falls between the global extension produced by uniformly distributed loads and the directional extension induced by localized loads, but it more closely resembles failure patterns triggered by localized loading.

At the final failure stage, all three types of slopes exhibit a fully developed slip surface and overall instability of the sliding mass. Under uniformly distributed loading, the slip surface penetrates deeply and continuously, producing large‑scale downslope displacement. Under localized loading, the shear surface typically shows three‑dimensional wedge‑shaped or tongue‑shaped propagation. The pushing‑type failure identified in this study is characterized by a distinct rear scarp, a bulging shear‑out zone at the slope front, and a through‑going slip surface that develops from top to bottom, ultimately forming an integrated sliding accumulation mass. Although this failure mode aligns more closely with the directional failure induced by localized loading, its progressive evolution is more complex, exhibiting a multi‑stage coupled sequence: initiation of rear‑edge tensile cracking → sliding of the upper‑middle block → frontal shear‑bulging extrusion → penetration of the slip surface → overall sliding.

Overall, the failure mechanism of colluvial slopes is jointly governed by the structural characteristics of the colluvial material, the looseness of joints, and the strong stress‑concentration effects induced by localized surcharge loading. In terms of failure evolution, the slopes exhibit both the “shallow‑to‑deep” shear‑extension pattern typical of uniformly distributed loading and the “point‑to‑surface” wedge‑shaped directional propagation associated with localized loading. However, the final evolution of colluvial landslides tends to develop into a typical pushing‑type failure, in which the failure process is more strongly influenced by the surcharge location, surcharge width, and the stratification of the colluvial deposits.

Therefore, the failure mechanism of colluvial slopes under surcharge loading cannot be simply categorized as either the uniformly distributed loading-induced mode or the localized loading-induced mode. Rather, it should be understood as a surcharge‑induced cumulative, pushing‑type, and structurally controlled failure mechanism that develops on the basis of the inherent structural weakness of the colluvial deposits.

**5.2.2 Comparison of Failure Mechanisms in Colluvial Slopes under Rainfall and Seismic Loading**

For the rainfall‑induced failure of colluvial deposits, the comparative reference used in this study is Ref. [16]. Khan et al. investigated the failure mechanism of unsaturated coal‑gangue colluvial slopes subjected to rainfall infiltration [16]. The failure process induced by rainfall infiltration in coal‑gangue slopes evolves through two distinct stages. The first stage is “seepage‑driven saturation and suction loss,” during which rainwater rapidly infiltrates through the highly permeable gangue material, causing matric suction to decrease sharply as the water content increases. Due to its highest permeability, the slope crest becomes saturated first, leading to nonuniform swelling and the formation of tensile cracks. The second stage is “strength degradation and initiation of sliding.” As seepage continues, pore‑water pressure shifts from negative to positive, effective stress progressively decreases, and the shear strength along the potential slip zone significantly deteriorates. Under the combined effects of self‑weight and seepage forces, a traction‑type sliding initiates from the crest and propagates downslope, ultimately reducing the factor of safety to below 0.9 and resulting in global slope failure.

For earthquake‑induced failures in colluvial deposits, Ref. [23] is used as the comparative source in this study. Ma et al. examined the failure mechanisms of colluvial slopes through shaking‑table experiments [23]. The seismic failure process of colluvial‑layer landslides can be summarized in two stages. The first stage is “crack initiation and propagation.” As seismic waves travel upward from the base of the slip zone, the combined effects of slip‑zone strength softening and topographic amplification lead to the formation of tensile cracks in the mid‑slope region. These cracks then propagate toward the slope toe, outlining the initial geometry of a throughgoing slip surface. The second stage is “slip‑surface formation and instability onset.” With continued shaking, the soil within the slip zone undergoes plastic yielding, the point safety factor decreases overall and exhibits a delayed peak, and a deep tensile crack forms at the rear of the sliding mass. The sliding mass subsequently undergoes a pushing‑type displacement from the mid‑slope toward the slope toe, ultimately resulting in global failure as the shear resistance along the slip zone drops sharply.

The failure mechanisms of colluvial slopes under different external disturbances exhibit both distinct differences and shared characteristics. Under rainfall conditions, landslide failure progresses through two stages: rainwater first infiltrates rapidly through highly permeable zones, causing early saturation at the slope crest and the development of tensile cracks; subsequently, rising pore‑water pressure leads to strength degradation along the slip zone, inducing traction‑type sliding from the crest toward the toe. Under seismic loading, failure initiates with tensile cracking in the mid‑slope region, after which the slip zone undergoes plastic yielding, and the sliding mass experiences a pushing‑type displacement from the mid‑slope toward the slope front. In contrast, slope failure induced by surcharge loading in this study exhibits a typical “pushing‑type” evolution. Surcharge‑driven stress redistribution leads sequentially to rear‑edge tensile cracking, crest sliding, and frontal bulging, during which the potential slip surface progressively develops and ultimately forms a tongue‑shaped sliding mass.

From a mechanistic perspective, rainfall‑induced landslides are primarily controlled by increases in pore‑water pressure and the degradation of shear strength, whereas earthquake‑induced landslides are driven by instantaneous shearing and plastic yielding. In contrast, surcharge‑induced landslides are triggered by stress accumulation and localized stress concentration, exhibiting progressive failure with pronounced structural‑control features. The failure process under surcharge loading involves both the “shallow‑to‑deep” shear propagation characteristic of uniformly distributed loads and the wedge‑shaped “point‑to‑surface” propagation induced by localized loads, while more prominently displaying a multistage interaction consisting of rear‑edge tensile cracking, translational sliding of the upper slope, and frontal bulging. Therefore, the failure mechanism of colluvial slopes subjected to surcharge loading is unique: it differs from the traction‑type sliding induced by rainfall and from the pushing‑type instability caused by earthquakes, instead manifesting as a cumulative-pushing-structurally controlled composite failure mode.

**5.2.3 Failure Mechanisms in Comparison with Rock Slopes**

In addition, colluvial slopes differ markedly from rock slopes, with their failure patterns more strongly governed by material weakness and structural looseness. Because rock masses possess higher structural integrity and more regular joint-fracture networks, the failure of rock slopes is typically controlled by geometrically well‑defined combinations of structural planes, resulting in structural instability modes such as block sliding, wedge failure, or toppling, with the failure surface being relatively predictable [59]. In contrast, colluvial slopes consist of rock fragments, soil materials, and loose accumulations, and their internal structure is highly heterogeneous. Their deformation generally develops through progressive plastic propagation, and their sliding surfaces tend to be more tortuous, less continuous, and more susceptible to local disturbances. Compared with the “structure‑controlled overall sliding” commonly observed in rock‑slope failures, colluvial slopes more readily initiate microcracks at sites of local stress concentration and progressively evolve along weak, poorly persistent zones under sustained surcharge loading, ultimately forming a pushing‑type overall slide.

Meanwhile, the differing responses of rock slopes under uniformly distributed loading and local loading are mainly reflected in the extent to which structural planes participate in deformation and in the geometric variations of shear bands. In contrast, because particle contacts within colluvial slopes are inherently loose, local surcharge loading more readily induces pronounced stress‑concentration effects, resulting in coupled deformation characterized by rear‑edge tension cracking, front‑edge bulging, and segmented displacement of the sliding mass. This leads to an overall evolutionary pattern of “zonal response-coupled failure” [59].

In summary, from an engineering perspective, colluvial slopes show pronounced failure sensitivity under surcharge loading. The progressive development of rear‑edge tension cracking, front‑edge bulging, and deep shear bands should be regarded as key monitoring indicators. Controlling surcharge intensity, limiting the spatial extent of surcharge loading, mitigating stress concentration at the surcharge-slope interface, and improving the shear strength of both shallow and deep layers of the colluvial deposits are essential measures for preventing surcharge‑induced landslides. In landslide‑prone regions, dynamic monitoring systems can be employed for continuous observation [60]."

6. Conclusions:

Ensure that the statements in this section accurately reflect the extent of the research and review conducted by the authors. Avoid broad generalizations.

Thanks for your comments. We have completed the revisions and have additionally incorporated the limitations of this study.

"**6.Conclusion**

This study aims to systematically elucidate the deformation‑response characteristics and failure mechanisms of colluvial slopes subjected to surcharge loading. By integrating physical model tests with FLAC3D numerical simulations, the study provides a comprehensive investigation of surcharge load transfer, stress distribution, deformation paths, and failure‑process evolution. Furthermore, a multivariate regression‑based similarity‑material proportioning method is proposed, offering systematic theoretical and methodological support for stability analyses of colluvial slopes under artificial loading. The main conclusions are as follows:

(1)Under surcharge loading, the maximum vertical displacement of 40 mm occurs at the slope crest, while the maximum horizontal displacement of 50 mm develops in the steeper mid‑slope region. The influence depth of vertical stress is substantially greater than that of horizontal stress, and the maximum shear stress is concentrated along the edge of the surcharge zone. The vertical stress beneath the surcharge area exhibits a depth‑dependent attenuation pattern.

(2)As the surcharge load increases, both the extent and intensity of regions exhibiting large shear‑strain increments expand, and the shear band progressively propagates from the slope crest toward the slope toe. The shear‑failure zones that form within the slope beneath the edge of the surcharge area and at the slope toe tend to extend toward the mid‑slope region.

(3)Under the same surcharge load, zones exhibiting pronounced shear‑strain increments are more likely to form closer to the slope‑break line. At a given surcharge location, a larger load leads to a greater extent of shear‑strain‑increment zone propagation toward the slope toe.

(4)The failure mode of accumulation‑slope masses under surcharge loading differs from that of homogeneous‑slope landslides and those induced by other loading conditions. The formation and evolutionary mechanism of surcharge‑triggered landslides proceeds as follows: surcharge application → disturbance of the initial equilibrium → initiation of rear‑edge tensile cracking → sliding of the upper soil mass → frontal compression and bulging → propagation and coalescence of the sliding surface → overall sliding failure.

(5)Accumulation slopes exhibit marked failure sensitivity under surcharge loading, and the progressive development of rear‑edge tensile cracking, frontal bulging, and deep‑seated shear zones should be regarded as key indicators for monitoring.

Due to limitations in the experimental conditions, this study still has several constraints:
(1) The experiment adopted only a static, staged surcharge‑loading scheme and did not consider influences such as rapid load application during construction, mechanical vibration, or traffic‑induced dynamic loading.(2) To simplify the computations, the numerical model employed the Mohr-Coulomb constitutive relation and did not incorporate mesoscopic effects such as structural‑plane weakening or particle breakage, which may cause the simulation results to be somewhat conservative or idealized.

Future research can be further advanced in the following directions:(1) Incorporating high‑precision in situ monitoring data to establish a multi‑scale validation framework that integrates experimental, numerical, and field observations.(2) Investigating mesoscopic failure mechanisms using discrete‑element or particle‑flow methods to clarify the fundamental influences of particle gradation and structural characteristics.(3) Introducing dynamic loading and unloading effects to simulate surcharge‑induced disturbance responses under realistic construction conditions.(4) Conducting multi‑field coupling studies involving surcharge loading, rainfall, seepage, and seismic actions to develop a comprehensive stability‑assessment framework for more complex environmental settings."

Thank you again and best wishes to you!

Sincerely yours,

Jiali Feng
